# Characterization of the *Escherichia coli* pyridoxal 5′‐phosphate homeostasis protein (YggS): Role of lysine residues in PLP binding and protein stability

**DOI:** 10.1002/pro.4471

**Published:** 2022-10-26

**Authors:** Angela Tramonti, Mohini S. Ghatge, Jill T. Babor, Faik N. Musayev, Martino Luigi di Salvo, Anna Barile, Gianni Colotti, Alessandra Giorgi, Steven D. Paredes, Akua K. Donkor, Mohammed H. Al Mughram, Valérie de Crécy‐Lagard, Martin K. Safo, Roberto Contestabile

**Affiliations:** ^1^ Istituto di Biologia e Patologia Molecolari Consiglio Nazionale delle Ricerche Rome Italy; ^2^ Istituto Pasteur Italia‐Fondazione Cenci Bolognetti and Dipartimento di Scienze Biochimiche “A. Rossi Fanelli” Sapienza Università di Roma Rome Italy; ^3^ Institute for Structural Biology, Drug Discovery and Development, Department of Medicinal Chemistry Virginia Commonwealth University Richmond Virginia USA; ^4^ Department of Microbiology and Cell Science University of Florida Gainsville Florida USA; ^5^ Genetics Institute University of Florida Gainesville Florida USA

**Keywords:** COG0325 family, PLPHP, pyridoxal 5′‐phosphate, vitamin B_6_, YggS

## Abstract

The pyridoxal 5′‐phosphate (PLP) homeostasis protein (PLPHP) is a ubiquitous member of the COG0325 family with apparently no catalytic activity. Although the actual cellular role of this protein is unknown, it has been observed that mutations of the PLPHP encoding gene affect the activity of PLP‐dependent enzymes, B_6_ vitamers and amino acid levels. Here we report a detailed characterization of the *Escherichia coli* ortholog of PLPHP (YggS) with respect to its PLP binding and transfer properties, stability, and structure. YggS binds PLP very tightly and is able to slowly transfer it to a model PLP‐dependent enzyme, serine hydroxymethyltransferase. PLP binding to YggS elicits a conformational/flexibility change in the protein structure that is detectable in solution but not in crystals. We serendipitously discovered that the K36A variant of YggS, affecting the lysine residue that binds PLP at the active site, is able to bind PLP covalently. This observation led us to recognize that a number of lysine residues, located at the entrance of the active site, can replace Lys36 in its PLP binding role. These lysines form a cluster of charged residues that affect protein stability and conformation, playing an important role in PLP binding and possibly in YggS function.

## Abstract 2

PDB Code(s): 7U9C, 7U9H, 7UAT, 7UAU, 7UAX, 7UBP, 7UBA, 7UB8 and 7UBQ;

**Abbreviations**
PLP: pyridoxal 5′‐phosphate
PLPHP: PLP homeostasis protein
PNP: pyridoxine 5′‐phosphate

## 1 INTRODUCTION

Pyridoxal 5′‐phosphate (PLP) is one of the six interconvertible vitamin B_6_ species: pyridoxal (PL), pyridoxine (PN), pyridoxamine (PM), and their respective 5′‐phosphate forms, namely, PLP, PNP, and PMP. Enzymes utilizing PLP as a cofactor (PLP‐dependent enzymes) catalyze a wide variety of biochemical reactions mainly involving amino acid substrates.^1^ Around 1.5% of the prokaryotic genes encode PLP‐dependent proteins, underlining the importance of vitamin B_6_ for microbial metabolism.^2^ As PLP is a highly reactive molecule and is both labile and toxic,^3^ the concentration of free PLP in the cell is tightly regulated.^4^ How PLP is provided to PLP‐dependent enzymes while maintaining a low concentration in its free form remains unknown even in the best‐studied model organisms such as *Escherichia* coli.^3^, ^4^ Recent studies have shown that the PLP Homeostasis Protein (PLPHP), named YggS in *E*. *coli* and previously named PROSC in humans, a member of the COG0325 family, is somehow ubiquitously involved in PLP homeostasis.^5^, ^6^, ^7^ Various pleiotropic phenotypes of the *E*. *coli yggS* mutant deletion (∆*yggS*) have been reported, which are clearly linked to amino acid and vitamin B_6_ metabolism, reflecting an imbalance in cellular PLP homeostasis.^5^, ^6^, ^8^, ^9^, ^10^ The *E*. *coli* ∆*yggS* mutant accumulates the PLP precursor PNP and is sensitive to an excess of PN but not of PL.^6^ The PN toxicity phenotype is complemented by the expression of eukaryotic *yggS* orthologues, including human PLPHP.^7^ It is also suppressed by the presence of amino acids, specifically isoleucine, threonine, and leucine, suggesting that PLP‐dependent enzymes are affected.^6^ However, the actual role of YggS in PLP homeostasis has not emerged so far. Importantly, mutations of the gene encoding PLPHP in humans are responsible for a rare and severe form of epilepsy.^7^, ^11^, ^12^, ^13^, ^14^, ^15^, ^16^ This is clear evidence of the involvement of PLPHP in PLP homeostasis, since epilepsy is mainly caused by an imbalance of the neurotransmitters, and several PLP‐dependent enzymes are involved in neurotransmitter metabolism.^3^, ^17^ Many of the above‐mentioned mutations affect amino acid residues of PLPHP located at the active site, and therefore decrease the affinity of the protein for PLP; others concern residues playing a role in protein stability.^18^ Clearly the capability of PLPHP to bind PLP is linked to its pivotal function in vitamin B_6_ homeostasis. Further, a link between the cyanobacterial (*Synechococcus elongatus*) homolog of YggS, PipY, and the nitrogen regulatory network has been also studied.^19^, ^20^ The prokaryotic PLPHP proteins are single‐domain monomers, consisting exclusively of the TIM barrel domain. The reported crystal structures show a conserved and solvent‐exposed PLP binding active site, hinting at a possible PLP transfer role (PDB Ids: 1W8G, 1CT5, and 5NM8). All characterized PLPHP do not have any apparent enzyme activity,^5^, ^21^ indicating that the protein‐bound PLP has no catalytic function. Several models may be proposed for PLPHP function. It could be a shuttle protein that brings PLP to target enzymes through a direct channeling mechanism, in which a complex is formed between PLPHP and PLP‐dependent apoenzymes. It may protect PLP from the environment, sequestering it and at the same time constituting a reservoir of the cofactor for PLP‐enzymes that might tap into. Finally, it could be part of a novel regulatory network that controls PLP homeostasis and is yet to be discovered.

Here we present a detailed characterization of *E*. *coli* YggS with respect to its PLP binding, atomic structure, as well as PLP transfer properties. We have serendipitously discovered that the K36A variant of the protein, affecting the residue that binds PLP covalently, is still able to bind PLP. In vitro and in vivo investigations on the role of other lysine residues in PLP binding and stability highlight the importance of these residues in the PLP binding and in the cellular function of YggS.

## 2 RESULTS

### 2.1 Physicochemical and PLP binding properties of recombinant *E. coli* YggS

**FIGURE 1:**
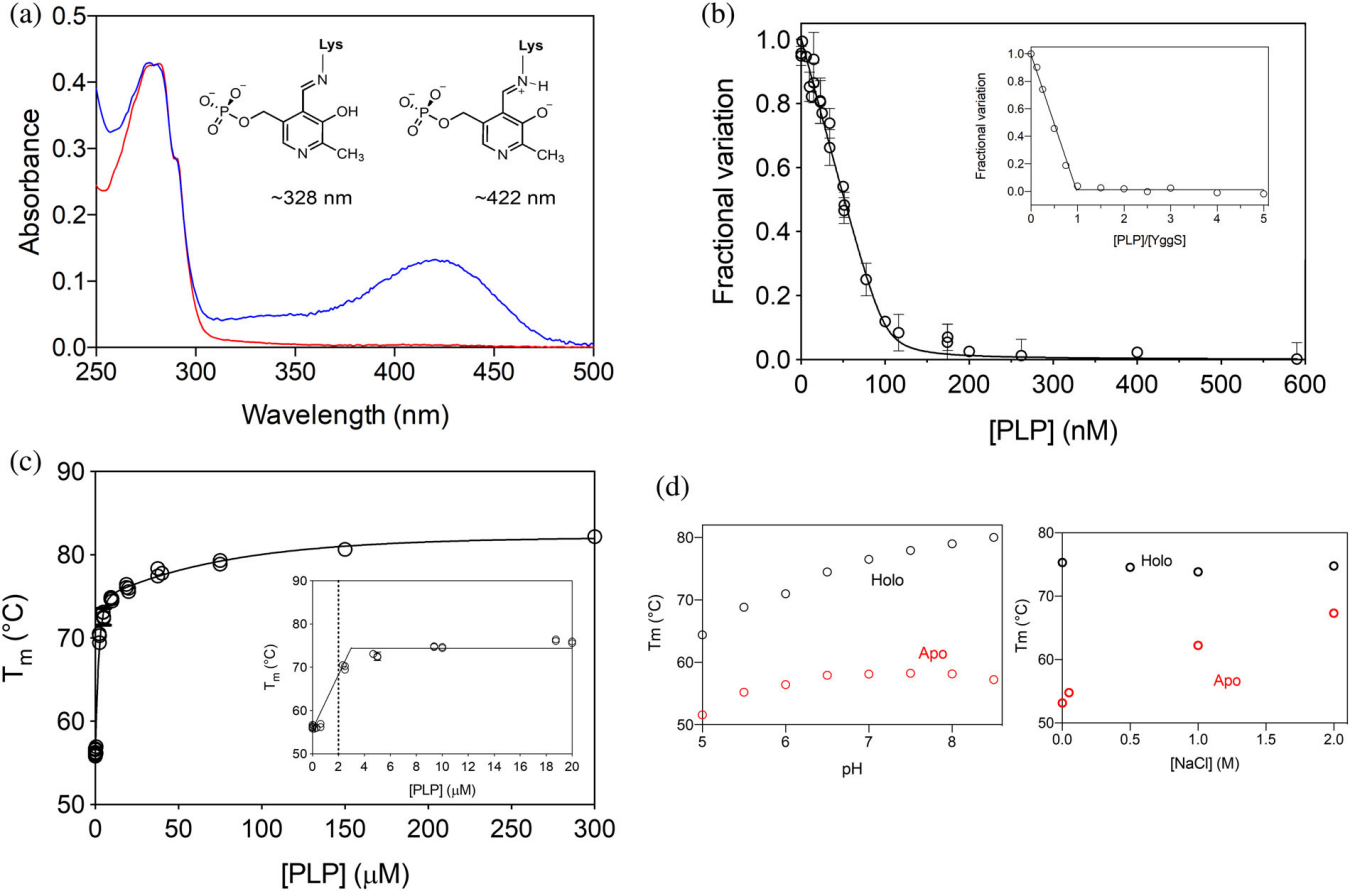
Properties of recombinant wild‐type YggS. (a) Absorption spectrum of apo‐ (red line) and holo‐YggS (blue line) measured in 50 mM NaHEPES buffer, pH 7.6. (b) PLP‐binding curves were obtained with fluorimetric measurements using 100 nM apo‐YggS in 50 mM NaHEPES, pH 7.6, upon excitation at 280 nm. Fluorescence change is expressed as fractional variation as a function of total PLP concentration and analyzed with Equation (1) described in Section 4. The inset shows the binding stoichiometry analysis obtained with 1 μM apo‐YggS. Fluorescence change, expressed as fractional variation as a function of the [PLPtot]/[protein] ratio, is linear as shown by the thick continuous line, up to the stoichiometry point corresponding to the crossing with the horizontal line. (c) Variation of the melting temperature of YggS, obtained by DSF analysis, as a function of the PLP concentration. Inset: zoom of the same saturation curve in the range 0–20 μM PLP. (d) Melting temperatures of apo‐ (red) and holo‐YggS (black) as a function of pH (left panel) and in the presence of different concentrations of NaCl (right panel)

The recombinant *E*. *coli* YggS purified to homogeneity was yellow in color. The absorption spectrum presented a maximum at 280 nm with two additional bands (Figure 1a). The major band at 422 nm corresponds to PLP covalently bound to the protein through a protonated Schiff base linkage, while the minor band at 328 nm corresponds to PLP bound in its enolimine form.^22^, ^23^ Based on the reported capability of YggS‐bound PLP to react with d‐ and l‐cysteine and form a thiazolidine adduct,^5^ a simple procedure was developed to obtain YggS in the apo‐form (Figure 1a; see Section 4 for details), which could be used to characterize the PLP binding properties of the protein. The kinetics of PLP binding to apo‐YggS, followed by measuring the absorbance increase due to the formation of the protonated Schiff base, was very fast with the binding equilibrium reached in the manual mixing time (Figure S1a). Equilibrium binding analyses were carried out by measuring the decrease of protein intrinsic fluorescence observed upon the addition of increasing PLP concentrations to a fixed concentration of apo‐YggS (Figure 1b). The obtained saturation curve was analyzed using the quadratic Equation (1), obtaining an estimated dissociation constant (*K*_D_) of 1.3 ± 0.8 nM (Table 1). The same procedure, using a higher protein concentration, allowed the titration of PLP binding sites, which returned a stoichiometry ratio of one PLP molecule per protein monomer (Figure 1b, inset). This stoichiometry was also confirmed by titrating the Schiff base formation detected by spectral changes observed upon the addition of PLP to 20 μM apo‐YggS (Figure S1a). Binding of other B_6_ vitamers (PL, PN, PNP) and of the vitamin B_6_ analogs 4‐deoxy‐PN (4dPN) and 4‐deoxy‐PNP (4dPNP) to YggS was also analyzed using the same fluorimetric method. A change in fluorescence emission, much smaller than that detected with PLP but clearly attributable to binding, could only be detected with PL and PNP (Figure S2), obtaining *K*_D_ values of 57 ± 20 μM and 31 ± 23 nM, respectively.

**TABLE 1:** Dissociation constant (*K*_D_) values of the PLP binding equilibrium, as determined by fluorescence change measurements

YggS forms	*K*_D_ (nM)
WT	1.3 ± 0.8
K137A	1.8 ± 1.9
ΔK233‐234	2.8 ± 0.7
K38A/K137A/K233A/K234A	23 ± 16
K36A/K38A	31 ± 20
K36A/K38A/K137A	43 ± 19
K36A/K137A	59 ± 10
K36A/K233A/K234A	91 ± 37
K36A	124 ± 19
K36A/K38A/K233A/K234A	N.D.
K36A/K137A/K233A/K234A	N.D.
K36A/K38A/K137A/K233A/K234A	N.D.

*Note*: Reported best‐fit values of K_D_ are expressed as the mean +/‐ the Standard Error of the Mean (SEM). N.D. Not determined. Fluorescence change was too small (or even absent) to allow determination of *K*_D_.

The thermal stability of holo‐ and apo‐YggS forms was analyzed by differential scanning fluorimetry (DSF). In 50 mM NaHEPES buffer at pH 7.6, binding of PLP to apo‐YggS has a significant stabilizing effect on the protein (Figure S3a). The addition of an equimolar amount of PLP to apo‐YggS increases the apparent *T*_m_ value from 56°C to 70°C, and increasing PLP concentration causes a further increase of *T*_m_, up to a value of about 82°C (Figure 1c, Table 2). This behavior suggests that two PLP molecules bind to the YggS monomer, although with different affinity. The molecule that binds with higher affinity evidently corresponds to PLP binding covalently at the active site of YggS and is also revealed by fluorimetric measurements. The lower affinity binding roughly corresponds to a *K*_D_ of 50 μM. This second PLP molecule does not bind covalently to the protein, as indicated by the lack of any change of absorbance at 420 nm when high concentrations of PLP are added to YggS (Figure S1b). Increasing pH from 6.0 to 8.5 has a stabilizing effect on both apo‐ and holo‐forms, whereas increasing NaCl concentration has a marked stabilizing effect only on the apo‐form, whose apparent *T*_m_ value approaches that of the holo‐form at 2 M NaCl (Figure 1d). DSF measurements were also employed to analyze the binding of other B_6_ vitamers and of B_6_ analogs to apo‐YggS, confirming that only PL and PNP bind, although it was not possible to determine *K*_D_ values using this technique, due to the small changes in *T*_m_ observed with such ligands (Figure S3b,c). It should be noted that, although PNP binds with relatively high affinity to apo‐YggS (31 ± 23 nM), it only marginally stabilizes the protein. Size exclusion chromatography (SEC) analyses of holo‐ and apo‐YggS showed that the protein exists as a monomer in both forms (Figure S4a). Interestingly, YggS samples purified without adding 2‐mercaptoethanol in the final dialysis step (see methods section), showed the presence of a minor fraction of dimeric YggS. This dimeric form was never present in fresh protein preparations obtained under reducing conditions (Figure S4b). These data were also supported by analytical ultracentrifuge (AUC) experiments with the recombinant YggS protein (Figure S4c).

**TABLE 2:** Apparent *T*_m_ values of YggS WT and variant forms, as determined by DSF

YggS form	*T*_m_ (°C)		
	Apo	Apo +2 μM PLP	Apo + 300 μM PLP
WT	55.1 ± 0.3	70.8 ± 0.8	82.2 ± 0.1
K137A	60.3 ± 0.1	71.5 ± 0.1	82.5 ± 0.1
ΔK233/K234	61.4 ± 0.8	73.1 ± 0.3	84.0 ± 0.4
K38A/K137A/K233A/K234A	63.4 ± 0.5	67.3 ± 1.2	80.7 ± 0.2
K36A	63.5 ± 0.2	64.6 ± 0.6	72.0 ± 0.3
K36A/K137A	64.8 ± 0.2	65.4 ± 0.2	72.7 ± 0.4
K36A/K233A/K234A	63.7 ± 0.1	63.7 ± 0.2	65.0 ± 0.1
K36A/K137A/K233A/K234A	64.6 ± 0.1	64.7 ± 0.1	66.0 ± 0.1
K36A/K38A	68.7 ± 0.1	69.0 ± 0.1	75.8 ± 0.3
K36A/K38A/K137A	68.9 ± 0.2	69.4 ± 0.1	75.2 ± 1.2
K36A/K38A/K233A/K234A	68.8 ± 0.1	68.9 ± 0.1	69.6 ± 0.1
K36A/K38A/K137A/K233A/K234A	68.7 ± 0.1	68.8 ± 0.1	69.1 ± 0.3

*Note*: According to the apparent *T*_m_ value of the apo‐form, the YggS variants were divided into three different categories corresponding to *T*_m_ intervals: (i) 55.1–63.4°C (in yellow); (ii) 63.5–64.8°C (in blue); (iii) 68.7–68.9°C (in green).

### 2.2 Crystal structure of *E. coli* YggS

#### 2.2.1 Overall structure

**FIGURE 2:**
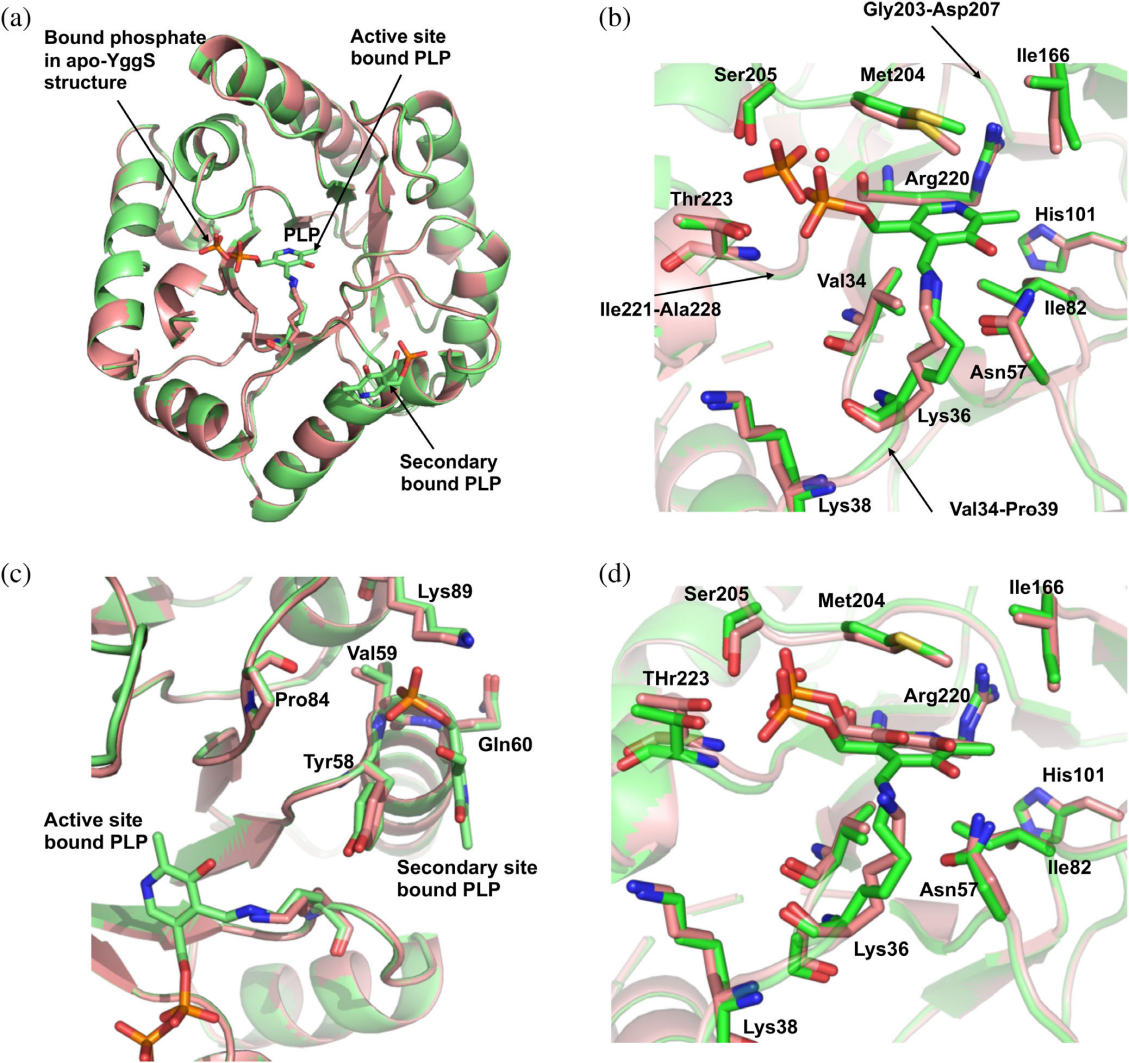
Crystal structure of WT YggS. (a) Superposition of holo‐YggS (green) with apo‐YggS (salmon). (b) Close‐up figure of the active sites (K36‐binding sites) of holo‐YggS (green) and apo‐YggS (salmon), the former with bound PLP. (c) Close‐up figure of the secondary PLP binding sites (K89‐binding sites) of holo‐YggS and apo‐YggS, the former with bound PLP. (d) Close‐up figure of the active sites of holo‐YggS (green) and YggS‐PNP (salmon)

The crystal structures of WT YggS in the apo‐form and in complex with either PLP (holo‐form or YggS‐PLP) or PNP (YggS‐PNP) have been determined to atomic resolutions ranging from 2.00 to 2.60 Å. Data collection and refinements are summarized in Table 3. All structures crystallized with one monomer in the asymmetric unit, and the crystal packing did not show any higher oligomerization consistent with the SEC and AUC analyses that suggest a functional monomeric protein. As previously described for PLPHP from *Saccharomyces cerevisiae* (PDB 1CT5) and *Synechococcus elongatus* (PDB 5NM8),^20^, ^21^ the structure folds as a TIM barrel,^24^ comprising eight α‐helices and eight β‐strands, and the holo‐form (YggS‐PLP) shows PLP at the active site, covalently bound through a Schiff base linkage to Lys36 (Figure 2a,b). Similarly, wild‐type crystal structures reported by others (1W8G from *E*. *coli*; 1CT5 from yeast; and 5NM8 from *Synechococcus elongatus*) also show that PLP present at the active site is covalently bound to Lys36 residue, which is conserved in all COG0523 proteins so far characterized.^21^ However, unlike the previous YggS structures, we also observed a bound PLP at a secondary site (Figure 2a,c). The presence of this second PLP molecule is evidently due to the high exogenous PLP concentration present in the crystallization conditions. In the YggS‐PNP structure, PNP is non‐covalently bound at the protein active site with similar binding pose as PLP (Figure 2d). The sequence identity between YggS from *E*. *coli* and *S*. *cerevisiae* or *S*. *elongatus* is ~32%, and superposition of our holo‐YggS structure with 1W8G from *E*. *coli*, 1CT5 from *S*. *cerevisiae*, and 5NM8 from *S*. *elongatus* yields rmsd of 0.1, 0.8, and 1.2 Å, respectively (Figure S5a). Comparison of monomeric apo‐YggS, YggS‐PLP, and YggS‐PNP structures also resulted in a very low rmsd of 0.1–0.2 Å. Nonetheless, there appear to be some localized structural changes, albeit small at the PLP binding site and the C‐termini of the structures; the latter is characterized by weak and missing electron density beyond Ala228, resulting in excluding K233 and K234 from the refined structures. Concerning this specific aspect, it should be noticed that although YggS is structurally similar to fold‐type III PLP‐dependent enzymes, whose prototype is alanine racemase, it lacks of the characteristic C‐terminal domain that in these proteins is responsible for the formation of a homodimer and forms part of the active site. As a consequence, in these enzymes, PLP is shielded by this two‐domain architecture and by the other subunit and has a limited accessibility from the solvent.^25^ In contrast, in the COG0325 family, PLP at the active site is much more exposed to solvent^20^, ^21^ and only the disordered C‐terminal end (comprising the K233 and K234 residues) may be capable of limiting exposure.

**TABLE 3:** Crystallographic data and refinement statistics for WT and variant YggS proteins with or without ligands

Data collection statistics	Holo‐WT	Apo‐WT	K36A	K137A	K36A/K38A	K36A/K137A	K36A/K38A/K233A/K234A	K38A/K137A/K233A/K234A	WT‐PNP
PDB ID	7U9C	7U9H	7UAT	7UAU	7UAX	7UBP	7UBA	7UB8	7UBQ
Space group	I4_1_22	I4_1_22	I4_1_22	I4_1_22	I4_1_22	I4_1_22	I4_1_22	P4_1_2_1_2	P4_1_2_1_2
Unit cell (Ǻ)	124.19	124.16	124.01	124.80	123.84	124.51	124.81	129.65	128.36
	124.19	124.16	124.01	124.80	123.84	124.51	124.81	129.65	128.38
	83.12	82.89	83.44	82.32	83.29	82.90	83.09	76.91	42.45
Monomer/AU	1	1	1	1	1	1	1	2	1
Resolution (Ǻ)	29.27–2.10 (2.16–2.10)	28.50–2.00 (2.05–2.00)	28.57–2.00 (2.05–2.00)	28.49–2.10 (2.16–2.10)	29.19–2.07 (2.13–2.07)	28.55–2.30 (2.38–2.30)	28.44–2.40 (2.49–2.40)	29.46–2.30 (2.38–2.30)	28.70–2.60 (2.72–2.60)
Unique reflection	19,252 (1557)	22,173 (1608)	22,247 (1601)	19,262 (1559)	20,017 (1538)	14,772 (1423)	13,080 (1360)	29,721 (2863)	11,385 (1354)
Redundancy	13.8 (14.0)	15.0 (14.0)	10.6 (10.6)	15.2 (15.2)	12.5 (12.1)	12.7 (12.8)	11.3 (11.7)	13.3 (13.6)	4.3 (4.4)
Average I/σ(I)	30.4 (4.7)	33.0 (4.2)	28.0 (5.1)	31.7 (5.7)	17.2 (2.9)	31.0 (8.6)	33.7 (8.5)	22.0 (7.7)	10.7 (2.9)
Completeness (%)	100 (100)	100 (100)	100 (100)	100 (100)	100 (100)	99.9 (100)	99.5 (100)	100 (100)	99.6 (100)
*R*_merge_ (%)^a^	6.9 (68.4)	6.8 (85.3)	5.0 (5.8)	7.3 (57.9)	9.6 (96.5)	5.6 (33.1)	5.6 (33.9)	8.8 (38.7)	14.0 (59.0)

Refinement statistics	Holo‐WT	Apo‐WT	K36A	K137A	K36A/K38A	K36A/K137A	K36A/K38A/K233A/K234A	K38A/K137A/K233A/K234A	WT‐PNP
Resolution (Ǻ)	28.54–2.10 (2.18–2.10)	28.50–2.00 (2.07–2.00)	28.57–2.00 (2.07–2.00)	28.41–2.00 (2.18–2.10)	29.19–2.07 (2.14–2.07)	28.37–2.30 (2.38–2.30)	27.91–2.40 (2.49–2.40)	28.99–2.30 (2.38–2.30)	28.70–2.60 (2.69–2.60)
No. of reflections	19,240 (1874)	22,162 (2205)	22,237 (2186)	19,251 (1899)	20,007 (1954)	14,762 (1449)	13,072 (1286)	29,668 (2903)	11,358 (1105)
*R*_work_ (%)	17.8 (23.5)	18.63 (23.0)	19.1 (23.8)	19.5 (26.0)	18.6 (26.3)	18.5 (25.7)	20.51 (26.80)	18.64 (20.90)	17.64 (22.96)
*R*_free_ (%)^b^	21.8 (26.9)	21.8 (28.9)	23.6 (30.5)	22.9 (26.2)	23.5 (30.1)	24.5 (31.4)	25.98 (34.99)	23.17 (25.53)	25.07 (27.21)
RMSD bonds (Ǻ)	0.008	0.008	0.008	0.007	0.008	0.007	0.008	0.008	0.007
RMSD angles (°)	1.20	1.17	1.16	1.16	1.17	1.17	1.21	1.19	1.16
Rama most favored (%)	97.3	97.3	97.8	97.3	96.9	97.8	96.0	97.3	98.2
Rama allowed (%)	2.7	2.7	2.2	2.2	3.1	2.2	4.0	2.7	1.8
Ave. B‐factor all atoms (Å^2^)	35.0	32.5	35.2	31.5	35.6	36.0	33.2	26.1	26.4
Ave. B‐factors protein (Å^2^)	33.73	31.46	33.89	30.65	34.65	34.96	32.49	25.99	26.26
Ave. B‐factors ligand (Å^2^)	56.36	55.64	64.84	48.74	74.86	66.80	73.69	31.50	30.17
Ave. B‐factors solvent (Å^2^)	42.25	39.01	42.92	37.73	42.58	40.47	31.94	27.08	27.44

^a^ *R*_merge_ = ∑_*hkl*_∑_i_|*I*_i_(*hkl*) − <*I*(*hkl*)>|/∑_*hkl*_∑_i_ *I*_i_(*hkl*).

^b^ R_free_ was calculated from 5% randomly selected reflection for cross‐validation. All other measured reflections were used during refinement.

In relation to the fluorescence emission quenching observed upon the addition of PLP to apo‐YggS, it may be assumed that, since the protein has only three tyrosine residues and two tryptophan residues, the emission obtained by exciting at 280 nm is mainly due to Trp residues.^26^ The observed fluorescence change may be correlated with Trp99 located at the base of the TIM barrel, about 15 Å from the PLP binding site. In fact, Trp99 has a potential cation‐pi stacking with His101 and both these residues are H‐bonded to Glu78. This arrangement is likely very sensitive to solvent exposure. On the other hand, Trp77 is located in a hydrophobic pocket and not likely to be sensitive to binding, although the observed quenching effect may also be due to the absorbance of light by PLP emitted by aromatic residues in the 330–340 nm region.

#### 2.2.2 Covalent binding of PLP at the K36‐binding pocket

Residues forming the PLP binding pocket or active site, (also referred to as K36‐binding pocket), include Leu32, Ala33, Val34, Ser35, Lys38, Gly55, Glu56, Asn57, Tyr58, Ile82, G83, Pro84, Met164, Ile166, Gly203, Met204, Ser205, Arg220, Ile221, Gly222, and Thr223. These residues, as well as specific PLP–protein interactions, are mostly conserved across the YggS homolog species (Figure S6).^21^ In addition to the Schiff‐base interaction between the aldehyde of PLP and the amine of Lys36, the O3, and N1 of PLP make hydrogen‐bond interactions with ND2 of Asn57 and NE of Arg220, respectively. The pyridine ring is sandwiched between Ala34, Ile82, and Met204, making extensive hydrophobic interactions with the side chains of these three residues. Other hydrophobic interaction involves C2A of PLP and Ile166. The phosphate group is involved in hydrogen‐bond interactions with the N and OG1 of Thr223, direct and/or water‐mediated interactions with N and OG of Ser205.

Comparison of the K36‐binding pocket of the holo‐YggS and the apo‐YggS shows that binding of PLP leads to subtle but significant structural changes at the K36‐binding pocket, especially the loop residues from Ser35 to Pro39 that have moved about 0.6 Å to allow the covalent interaction between PLP and K36 (Figure 2a,b). The side‐chains of the other binding pocket residues have also moved slightly to optimize their interactions with PLP. It is notable that even though apo‐YggS showed no presence of PLP at the K36‐binding pocket, we do observe a bound phosphate or sulfate (likely from the buffer and/or precipitant used to purify or crystallize the protein) close to the bound PLP phosphate in the holo‐structure; the two separated by 2.7 Å between the phosphorous atoms (Figure 2).

#### 2.2.3 Non‐covalent binding of PLP at a secondary site

Quite interestingly, and not previously reported, we do observe a second PLP bound at a surface cleft defined by the conserved residues Lys89, Gln60, Val59, Pro84, Tyr58, and Val59 (henceforth referred to as K89‐binding pocket; Figure 2c); at a distance of ~14 Å from the K36‐binding pocket. The phosphate group makes direct hydrogen‐bond interactions with NZ of Lys89, N of Gln60 and Val59, and water‐mediated hydrogen‐bond interaction with O of Pro84. The PLP pyridine ring is stacked parallel against Tyr58 ring, making extensive π–π interactions. These interactions, expectedly, should add to the stability of the protein. The non‐covalent binding of PLP to a secondary site is consistent with the DSF results, obtained by us with a very high PLP concentration (Figure 1c).

The second bound PLP molecule, unlike the active‐site bound PLP, shows significantly weaker density, which is manifested in high B factors of the PLP atoms. In some of the structures, the PLP density was so weak that a phosphate or sulfate molecule was fitted into the density and refined. We should point out that, in the apo‐YggS (where PLP is completely removed from the protein), we only observed bound phosphate from the buffer, while in YggS‐PNP complex, where PLP is completely removed and the protein (in Hepes buffer) crystallized in the absence of sulfate or phosphate, as expected we do not observe any bound phosphate/sulfate at the secondary site.

#### 2.2.4 Non‐covalent binding of PNP at the K36‐binding pocket

When PNP was added to apo‐YggS during crystallization, we observed a non‐covalently bound PNP molecule at the K36‐binding pocket, with similar orientation and binding interactions as described above for the non‐covalently bound PLP (Figure 2d). The lack of Schiff‐base interaction with K36 explains the relatively low affinity of YggS for PNP compared to PLP (Figure S2b).

### 2.3 The K36A YggS mutant binds PLP covalently as a Schiff base

**FIGURE 3:**
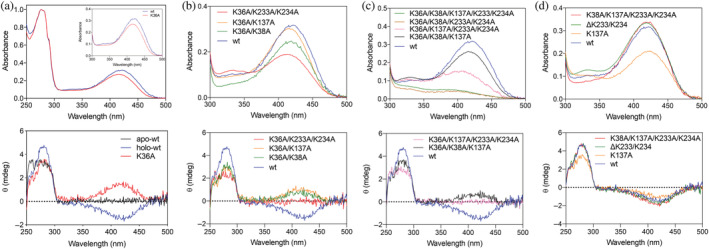
Spectroscopic properties of lysine variants of YggS. Absorption (upper panels) and CD spectra (lower panels) of (a) K36A single variant; (b) K36A/K38A, K36A/K137A, and K36A/K233A/K234A double variants; (c) K36A/K38A/K137A, K36A/K38A/K233A/K234A, K36A/K137A/K233A/K234A triple and K36A/K38A/K137A/K233A/K234A quadruple variants; (d) K38A/K137A/K233A/K234A, K137A and ΔK233/K234 K36‐containing variants. Spectra of WT‐YggS (in blue) is reported in all panels as reference

As noted above, the crystal structure of wild‐type (WT) YggS shows PLP covalently bound to a conserved Lys36 residue.^21^ Ito et al.^5^ previously reported that purified K36A YggS variant protein lacked the characteristic 420 nm absorption band, suggesting that PLP cannot be covalently bound when K36 is replaced with a residue that lacks a primary amino group. However, when we expressed and purified the K36A YggS variant to homogeneity, we observed an absorption spectrum with a band with maximum at 416 nm that suggests the presence of a protonated Schiff base linkage between PLP and the protein (Figure 3a upper panel). Deconvolution of WT and K36A absorption spectra into the related component absorption bands shows that the variant protein presents a less intense contribution at 420 nm and a 390 nm component band, instead of the 375 nm band present in the WT protein (Figure S7). In order to ascertain whether PLP in the K36A variant is actually covalently bound to the protein through a Schiff base linkage, we carried out an experiment in which both the WT and K36A proteins (60 μM) were treated with 8 mM NaBH_4_. The treatment causes the reduction of the Schiff base linkage that irreversibly anchors the cofactor to the protein.^27^ Conversely, aldehyde PLP is reduced to pyridoxine 5′‐phosphate that is not covalently bound to the protein. Treatment with NaBH_4_ resulted in the disappearance of the 416–420 nm bands, accompanied by the formation of a ≈315 nm band. Samples were then denatured by the addition of 0.2 M NaOH and concentrated in centrifuge filters with cutoff of 10 kDa. The absorption spectra of the filtered samples, containing only small molecular weight molecules were recorded. Concentrated protein samples retained by the filter were diluted with HEPES buffer and concentrated repeatedly, so as to eliminate small molecular weight molecules. Figure S8 compares the absorption spectra of protein samples and small molecular weight samples. It is clear that both WT and K36A protein samples contain a reduced form of PLP (320 nm absorption bands), albeit present to a lesser extent in K36A, that evidently derives from PLP internal aldimine. On the other hand, a protein‐free form of the cofactor, evidently pyridoxine 5′‐phosphate (302–308 nm bands^28^) deriving from aldehyde PLP is present in both small molecular weight samples (to a greater extent in K36A). This mostly derives from an excess of PLP that we added to keep the proteins in the holo‐form but that we were not able to completely remove with a size exclusion chromatography. Noticeably, the K36A sample also contains a small 390 nm band, which corresponds to unreduced aldehyde PLP.

In contrast to WT YggS, which shows a negative 420 nm visible circular dichroism (CD) band, the K36A variant presents a positive 420 nm band (Figure 3a lower panel). The near UV region between 260 and 290 nm is also different in the spectra of the two YggS forms. Moreover, the K36A variant shows a lower affinity for PLP than the WT YggS (*K*_D_ = 124 ± 19 nM vs. 1.3 ± 0.8 nM; Table 1). DSF analysis of PLP binding to K36A YggS indicates the binding of two PLP molecules with very different affinity, a behavior that is similar to that of the WT protein, and shows that the lower affinity non‐covalent binding event is not affected by the K36A mutation (Figure S9).

The spectroscopic observations and NaBH_4_ reduction experiments indicate that PLP bound with higher affinity in K36A YggS is linked as a Schiff base (Figure 3a, upper panel and Figure S8) to alternative lysine residues and is either bound at the active site with a different orientation with respect to WT YggS or at another site (Figure 3a, lower panel), as suggested by the positive Cotton band in the K36A CD spectrum. This is quite an unusual finding, since many variant PLP‐dependent proteins whose active site lysine was replaced have been characterized, but none of them has been reported to bind PLP covalently to other lysine residues.^29^, ^30^, ^31^, ^32^ This observation suggests that, in the specific case of YggS, the presence of alternative lysine residues that are able to bind PLP covalently in the absence of K36 may be related to the function of the protein.

### 2.4 Covalent binding of PLP to YggS: The role of lysine residues other than Lys36

In order to identify the alternative lysine residue(s) that bind(s) PLP covalently in the K36A variant, a number of multiple lysine variants of YggS were produced and characterized, focusing on those residues that are located near the active site entrance (K38, K137, K233, and K234; see Figure S10). Our strategy was to start from the K36A variant and gradually add further variations so as to obtain all possible combinations of double, triple, and quadruple variants. Variations of the last two residues at the C‐terminal end of the protein, K233 and K234, were always introduced together and are therefore considered as a single K233A/K234A variation. The K36A/K38A and K36A/K137A double variants were able to bind PLP covalently as a Schiff base, as judged by their absorption spectra (Figure 3b upper panel) that, similarly to K36A, show a maximum absorbance at 416 nm and a more intense component band at 375 nm with respect to WT (Figure S7). Both variants also show a positive 420‐nm CD band (Figure 3b lower panel) that is somewhat less intense with respect to K36A, suggesting that PLP is bound covalently to the protein with similar modalities with respect to the K36A variant (Figure S7). On the other hand, the K36A/K233A/K234A variant shows a substantially different absorption spectrum (Figure 3b upper panel), with a less intense component band at 415 nm and more prominent component bands at 340 and 315 nm (Figure S7). Moreover, the CD spectrum of this variant does not show any band in the visible region (Figure 3b lower panel), suggesting that PLP may be linked to a different lysine residue with respect to the K36A variant. Then, we produced all possible triple variants (K36A/K38A/K137A, K36A/K137A/K233A/K234A; K36A/K38A/K233A/K234A) and a quadruple variant (K36A/K38A/K137A/K233A/K234A). We found that only the triple K36A/K38A/K233A/K234A and the quadruple K36A/K38A/K137A/K233A/K234A variants had lost the capability to bind PLP covalently, but were still able to bind PLP non‐covalently in its free aldehyde form (presumably at the active site), although with low affinity (as judged by the presence of a feeble 390‐nm absorption band; Figure 3c upper panel). The K36A/K137A/K233A/K234A triple variant shows absorption and CD properties very similar to the double K36A/K233A/234A variant (Figure 3c lower panel; Figure S7). The K36A/K38A/K137A variant shows a CD spectrum similar to K36A/K38A but an absorption spectrum with a less intense 415 nm component and more prominent 315 and 340 nm components. Finally, as control variants, a multiple K38A/K137A/K233A/K234A variant, in which all lysine residues were mutated except K36, and single K137A and ΔK233/K234 variants were produced. All these variants were able to bind PLP as a Schiff base and showed absorption and CD spectra very similar to those of WT YggS (Figure 3d; Figure S7).

Altogether, the spectral properties of YggS variants suggest that in the K36A variant PLP is most likely covalently bound to either K38 or K233/K234. Only the concomitant replacement of K36, K38, K233, and K234 to Ala, abolishes the capability to bind PLP covalently. Far UV‐CD spectra of all variants and/or crystallographic studies of several of the variants, including the triple variants (vide infra), demonstrated that the amino acid replacements did not affect the overall secondary structure of the protein (Figure S11). SEC analyses showed that all variant proteins, just like the WT YggS, were in the monomeric state.

PLP binding to YggS lysine variants was analyzed by fluorometric titration. The obtained *K*_D_ values indicate that K137A and ΔK233/K234 variants have high affinity for PLP, similarly to WT YggS (Table 1). Whereas, the K36A variant shows a 100‐fold higher *K*_D_ value. Interestingly, as further variations concerning lysine residues are added to the K36A variant, the *K*_D_ values are decreased. With the K38A/K137A/K233A/K234A variant, in which K36 is present but all other Lys residues are replaced by Ala residues, the *K*_D_ value is about 18‐fold higher than WT.

### 2.5 Assessing putative PLP attachment sites in recombinant YggS variants by mass spectrometry

**FIGURE 4:**
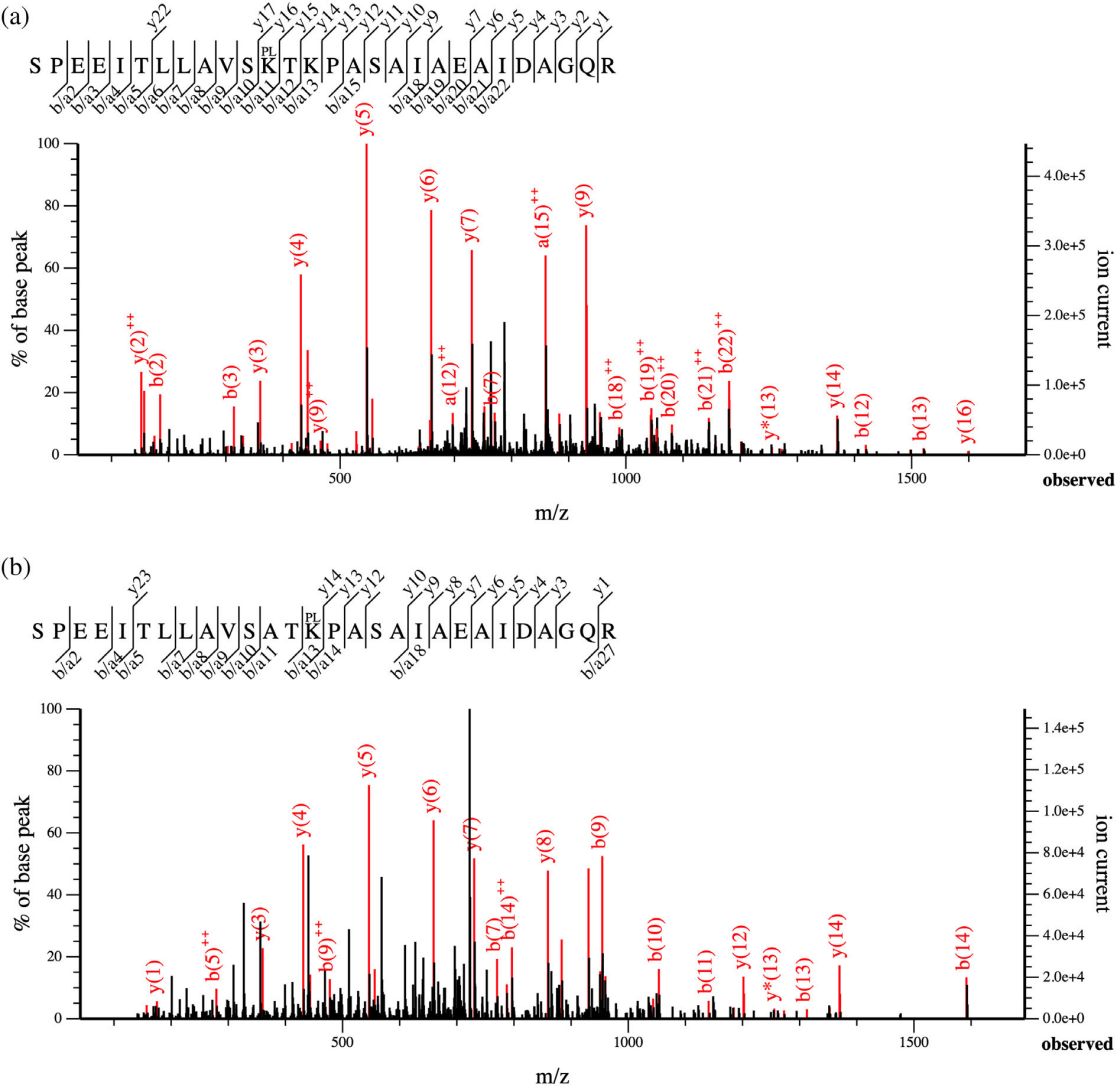
Fragments observed in the MSMS spectrum. (a) MSMS spectrum of the WT tryptic peptide SPEEITLLAVSK*TKPASAIAEAIDAGQR, *m*/*z* = 755.1628 and *z* = 4, with a mass shift of +151.06 Da at K*, corresponding to reduced PLP after phosphatase treatment. Mascot Score 73; Sequest Xcorr 11.54. (b) MSMS spectrum of the K36A tryptic peptide SPEEITLLAVSATK*PASAIAEAIDAGQR, *m*/*z* = 740.8958 and *z* = 4, with a mass shift of +151.06 Da at K*, corresponding to reduced PLP after phosphatase treatment. Mascot Score 60; Sequest Xcorr 8.15

The WT, K36A, and K36A/K38A proteins were analyzed by mass spectrometry to determine the identity of the alternative PLP‐binding lysine residues. Protein samples were treated with NaBH_4_, in order to reduce any Schiff base linkage between PLP and Lys residues, and irreversibly attach PLP to the protein before tryptic digestion. From the analysis of WT YggS, the trypsin‐specific peptide SPEEITLLAVSKTKPASAIAEAIDAGQR with a mass shift of +151.06 Da, corresponding to reduced PLP with the phosphate group removed (see Methods section for details), was identified with high confidence (Mascot score 73; Sequest HT XCorr 11.54; Figure 4), confirming covalent attachment of PLP, presumably to Lys36, as indicated from crystallographic data. Analysis of the K36A YggS variant identified the tryptic peptide SPEEITLLAVSATKPASAIAEAIDAGQR also with a mass shift of +151.06 Da (Mascot score 60; Sequest HT XCorr 8.15; Figure 4), suggesting Lys38 to be an alternative PLP binding residue when Lys36 is replaced by an Ala residue. No peptides from the K36A/K38A variant of YggS were found to be modified by reduced PLP.

Further, the whole protein mass of each protein was determined by LC–MS after treatment with sodium borohydride. The predominant form of the WT protein was found to be 27,035 Da, accompanied by a minor form with mass of 26,804 Da (data not shown). The difference of 231.1 Da corresponds well with the absence of PLP in the apo form. The predominant form of the K36A/K38A protein was found to be 26.8 kDa (data not shown). The predominant form of WT closely matches that expected for the full‐length protein plus PLP covalently bound, while the minor form is consistent with the apo‐form of the WT recombinant protein. For the K36A/K38A variant, the mass exactly matches that expected for the recombinant protein with no PLP covalently bound to it.

### 2.6 Lysine mutations confer stability to the YggS monomer

Interestingly, DSF analyses highlighted some important differences between WT and the lysine variants. According to the apparent *T*_m_ value of the apo‐form, the YggS variants may be divided into three different categories corresponding to the following *T*_m_ intervals: (i) 55.1–63.4°C (WT and variants in which K36 is present); (ii) 63.5–64.8°C (variants in which the K36A variation is present alone or in combination with K137A and K233A/K234A); (iii) 68.7–68.9°C (variants in which the K38A mutation is also included, in combination with K36A, K137A and K233A/K234A; Table 2). With all YggS forms belonging to category (i), the addition of an equimolar amount of PLP (that binds covalently to K36 at the active site) considerably increases the apparent *T*_m_ value (from 4 to 16°C) conferring stability to the protein against thermal denaturation. Interestingly, the *T*_m_ value of apo‐YggS protein variants in categories (ii) and (iii) is generally much higher than that of category (i) variants. Moreover, the addition of an equimolar amount of PLP has a very small effect, or no effect at all, on the *T*_m_ value of categories (ii) and (iii) variants. The crystal structure of YggS shows K36 in close proximity to R220 (Figure 2b), K65, and K38, although much closer to K38 (Figure S10). It also appears that even though the C‐terminus residues K233 and K234 are missing from the YggS structure, the location of the C‐terminus suggests that these two lysine residues would also cluster close to K36, K38, and K65 and R220 (Figure S10). Clearly, the close proximity of these positively charged lysine residues is expected to destabilize the protein. In particular, the close proximity of K36 and K38 in the structure should lead to a destabilizing effect as noted above but ameliorated when these two residues are mutated to Ala or when K36 binds PLP. Consistently, variants that include K36A are more stable and stability increases when K38A is also included. Covalent binding of PLP to K36 neutralizes some of the clustered positive charges, explaining the observed increase in *T*_m_ with WT YggS and variants that contain K36. This is not observed with variants in which K36 is replaced with an Ala residue. The addition of 300 μM PLP, which probably saturates the second binding site where PLP binds non‐covalently, distinct from the active site, determines a further increase of about 11°C in the *T*_m_ of category (i) variants. A smaller but consistent increase in *T*_m_ (about 7°C) is also observed in categories (ii) and (iii) variants in the presence of excess PLP, except in those where K233 and K234 are mutated, which show either a slight increase in *T*_m_ or no increase at all (Table 2). Binding of the second PLP adds to the stability of the protein, likely as a result of additional protein interactions mediated by PLP.

### 2.7 Role of lysine residues in the conformational state of YggS

**FIGURE 5:**
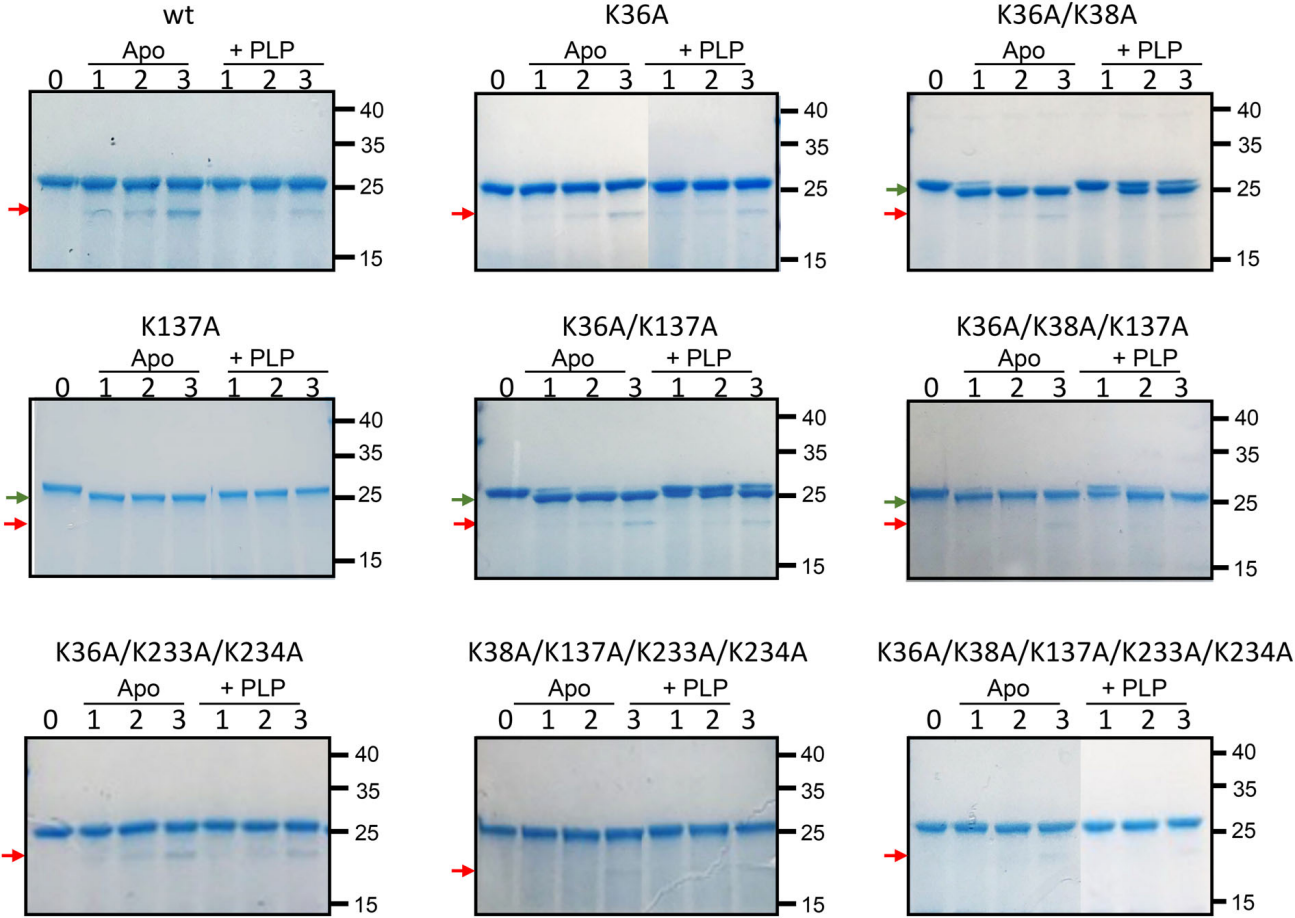
Limited proteolysis of YggS forms. SDS/PAGE analysis of YggS limited proteolysis time course, in the absence or presence of PLP. Apo‐forms of YggS (6.5 μM; the calculated molecular weight of the recombinant protein with the poly‐His tag is 26,853 Da) were exposed to trypsin digestion either in the presence (+PLP) or absence (Apo) of 20 μM PLP (see methods section for details). Arrows indicate the main fragments resulting from trypsin digestion (green arrow, 26 kDa band; red arrow, 21 kDa band; molecular weights were estimated using the MS Image Capture‐A software of the Major Science Digimage System), which were analyzed by mass spectrometry as explained in the text. Numbers on the right correspond to molecular weights (in kDa), as indicated by the protein molecular weight standard (PageRuler prestained protein ladder; ThermoFisher Scientific) that was used in the electrophoretic run, which for simplicity was not included in the figures. Some images were generated by splicing together different gels in order to allow the comparison of different data sets.

In order to analyze and compare the conformational status of WT and variant YggS forms, limited proteolysis experiments were carried out using trypsin. Upon 1‐hr incubation of the WT apo‐YggS with trypsin (see the experimental section for details), a new proteolysis band of around 21 kDa becomes visible (in addition to the 27 kDa band corresponding to the intact YggS protein) whose intensity increases with time (Figure 5). Mass spectrometry (MS) analysis of the new 21 kDa proteolytic fragment indicated that the tryptic cleavage took place at Arg175. It is clear that the addition of PLP to WT apo‐YggS slows down this proteolysis process (Figure 5), indicating that PLP binding could be inducing some protein conformational change and/or reducing flexibility and/or the accessibility of R175 to trypsin. Compared to WT apo‐YggS, trypsin cleavage is slower in the apo‐form of the K36A and K36A/K233A/K234A variants, and the addition of PLP does not have any effect on the rate of the proteolysis process. With the double K36A/K38A variant, another cleavage site is exposed to the action of trypsin, which generates a 26 kDa band. MS analysis of this fragment suggested as possible cleavage site Arg229. Cleavage in this area was confirmed by the observation that the double K36A/K38A variant in the apo‐form, incubated with trypsin for 3 hr, was not retained by a Ni‐NTA chromatography column, indicating that the polyHis tag placed at the C‐terminal end was missing. PLP binding has the effect to slow down the cleavage at this second site. This second trypsin cleavage site is also exposed in the K137A variant and in the K36A/K137A and K36A/K38A/K137A multiple variants. Analysis of the 26 kDa fragment generated by limited proteolysis of the K36A/K137A variant suggested that trypsin cleavage also in this case took place at Arg229. In all these variants, PLP binding neatly slows down the cleavage process. The K38A/K137A/K233A/K234A and K36A/K38A/K137A/K233A/K234A variants are both cleaved at Arg175 (that generates a proteolysis band of around 21 kDa); however, the cleavage process is much slower than that of all other variants and PLP does not have any visible effect on its rate. With variants containing the K233A/K234A mutation, the 26 kDa band is not generated by trypsin proteolysis. This observation suggests that mutation of residues Lys233 and Lys234 affects the exposure of Arg229 to trypsin cleavage.

### 2.8 Crystal structures of YggS lysine variants

The crystal structures of the YggS variants K36A, K137A, K36A/K137A, K36A/K38A, K36A/K38A/K233A/K234A, and K38A/K137A/K233A/K234A, have been determined to atomic resolutions ranging from 2.00 to 2.40 Å (Table 3). Also, in this case, all structures show one monomer in the asymmetric unit, with the exception of the K38A/K137A/K233A/K234A variant that crystallized with two monomers. Comparison of all structures reported here, whether WT or variants bound or unbound with PLP or PNP, resulted in a very low rmsd (0.1–0.2 Å), indicating very little if any significant overall structural changes between the structures. This observation is consistent with the far UV CD spectra of all protein forms demonstrating that the amino acid replacements did not affect the overall secondary structure of the protein (Figure S11).

#### 2.8.1 Non‐covalent binding of PLP at the K36‐binding pocket in YggS‐K36A variant

**FIGURE 6:**
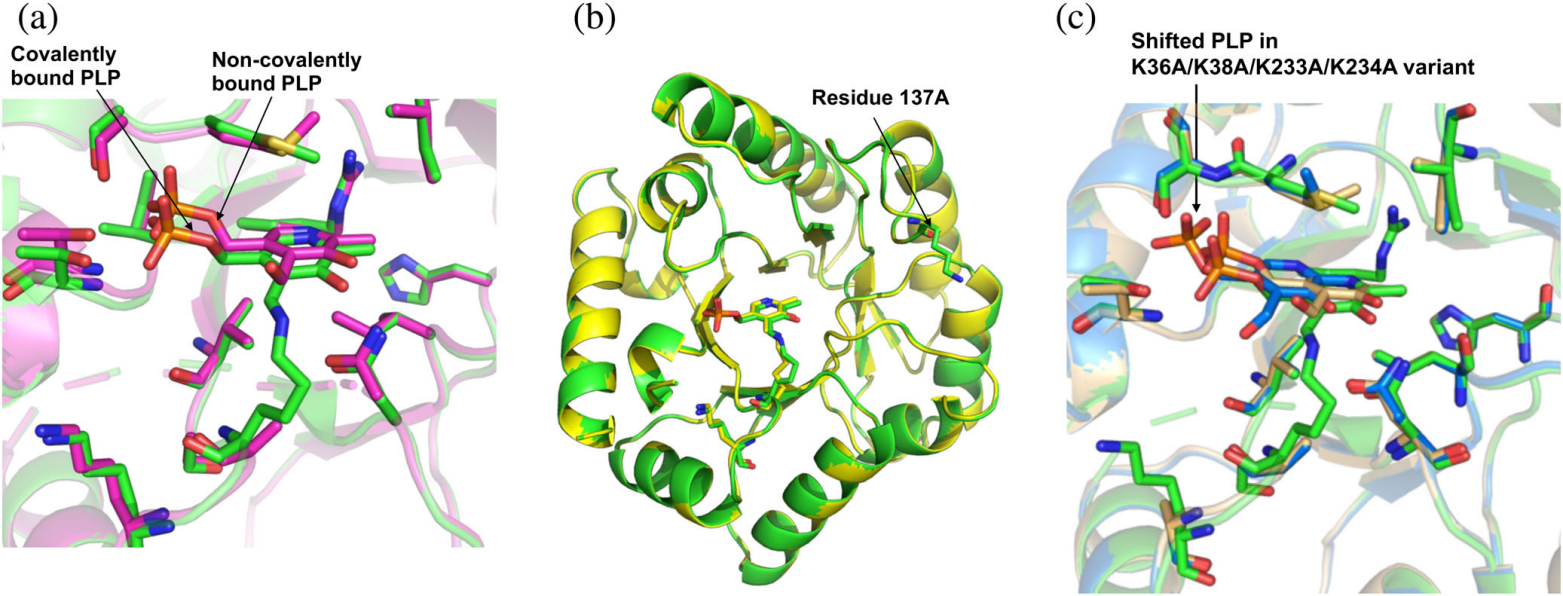
(a) Superposed close‐up figure of active site of holo‐YggS (green) and K36A YggS (magenta) showing covalently and non‐covalently bound PLP. (b) Superposition of holo‐YggS (green) with K137A YggS (yellow) showing the mutation site at residue 137. (c) Superposed close‐up view of the active site showing covalent PLP binding in holo‐YggS‐PLP (green), and non‐covalent binding in K36A/K137 (cyan) and K36A/K38A/K233A/K234A variants (amber)

The crystal structure of the YggS variant with Lys36 mutated to Ala does not show a Schiff‐base linkage with the aldehyde of PLP at the K36‐binding pocket. Nevertheless, and interestingly, PLP, whether co‐crystallized with the variants or picked up from the cell, still binds at the K36‐binding pocket although non‐covalently, occupying almost the same position and making similar non‐covalent protein interactions as the covalently bound PLP of WT YggS (Figure 6a). In the WT holo‐YggS, PLP is rotated ~0.7 Å with respect to the non‐covalently bound PLP in K36A YggS.

#### 2.8.2 Crystal structures of K137A, K36A/K137A, K36A/K38A, K36A/K38A/K233A/K234A, and K38A/K137A/K233A/K234A variants

The crystal structure of the K137A variant binds PLP covalently at the K36‐binding pocket while K36A/K137 binds PLP non‐covalently. The covalently bound PLP in K137A or non‐covalently bound PLP in K36A/K137A variant shows similar protein interactions as described above for the WT and the K36A variant, respectively. Mutation of Lys137, which is located on a loop and is exposed to the solvent, does not lead to any significant structural changes at the mutation site in both the K137A and K36A/K137 variants (Figure 6b).

Also, the K36A/K38A and K36A/K38A/K233A/K234A variants bind PLP non‐covalently at the K36‐binding pocket. The K36A/K38A variant shows strongly bound PLP with similar protein interactions as the non‐covalently bound PLP in the K36A/K137 variant. In the K36A/K38A/K233A/K234A variant, the non‐covalently bound PLP appears weakly bound, especially the pyridine ring, and shifted about 2.7 Å from the K36A/K38A PLP position (Figure 6c). Interestingly, the positional shift of the K36A/K38A/K233A/K234A variant PLP has resulted in the PLP phosphate overlapping the observed phosphate or sulfate molecule in the apo‐YggS structure (see above). The shift has also led to significant differences in PLP interactions with the protein. For example, in the K36A/K38A/K233A/K234A variant, the direct hydrogen‐bond interaction between the O3 or N1 of PLP with ND2 of Asn57 (observed in all the YggS structures) is now replaced by a water‐mediated interaction, while N1 of PLP which was observed to bind to NE of Arg220 now makes interaction with O of Arg220. The hydrophobic interactions between the pyridine ring, Ala34, and Met204, although maintained, show significantly different contacts. The phosphate group makes direct interaction with OG of Ser205 instead of water‐mediated as observed in the WT or the other YggS structures, while the hydrogen‐bond interaction between the phosphate and Thr225 side‐chain is now abrogated. The PLP position in the K36A/K38A/K233A/K234A variant could represent a snapshot of PLP movement into the active site.

Not surprisingly, PLP binds covalently to Lys36 in the K38A/K137A/K233A/K234A variant with very similar protein interactions as the WT. Like the K36A/K137A variant, we did not observe any significant structural changes at the K137 mutation site.

Although mass spec analysis suggests PLP binding to Lys38 in the K36A variant (Figure 4) and spectroscopic analyses indicate a Schiff base formation between PLP and the protein, such covalent binding does not appear in the present crystal structures. Lys38 is located near the active site and shielded by several residues; therefore, any covalent binding of PLP to Lys38 would require a significant change in the placement of PLP at the active site, in agreement with the change of CD spectrum observed in the K36A variant (Figure 3a). Evidently, such conformational species with bound PLP exist in solution but did not crystallize out.

### 2.9 In vitro interactions between YggS and the model PLP‐dependent enzyme SHMT

We conducted studies to determine whether there is an interaction between YggS and *E*. *coli* serine hydroxymethyltransferase (*e*SHMT). This enzyme was chosen as model PLP‐dependent enzyme since physical clustering associations linked YggS to the PLP‐dependent enzyme *e*SHMT encoded by the *glyA* gene. Moreover, a synthetic lethality phenotype was observed in the Δ*yggS* Δ*glyA* double mutant, which is not viable on LB medium.^33^ Surface Plasmon Resonance (SPR) assays were carried out to analyze the biophysical interaction between apo‐SHMT and holo‐YggS. Apo‐SHMT was immobilized on activated sensor chips via amine coupling. Experiments were carried out twice at several different holo‐YggS concentrations (0.24, 0.72, 2.2, 6.7, 20, 60, and 180 μM) at a constant flow (30 μl/min). SPR experiments showed that interactions between apo‐SHMT and holo‐YggS took place (Figure S12A). Scatchard analysis (Figure S12B) shows that holo‐YggS interacts with apo‐SHMT with a *K*_D_ of 4.8 ± 0.8 μM.

### 2.10 Kinetics of PLP transfer from WT holo‐YggS to apo‐SHMT

**FIGURE 7:**
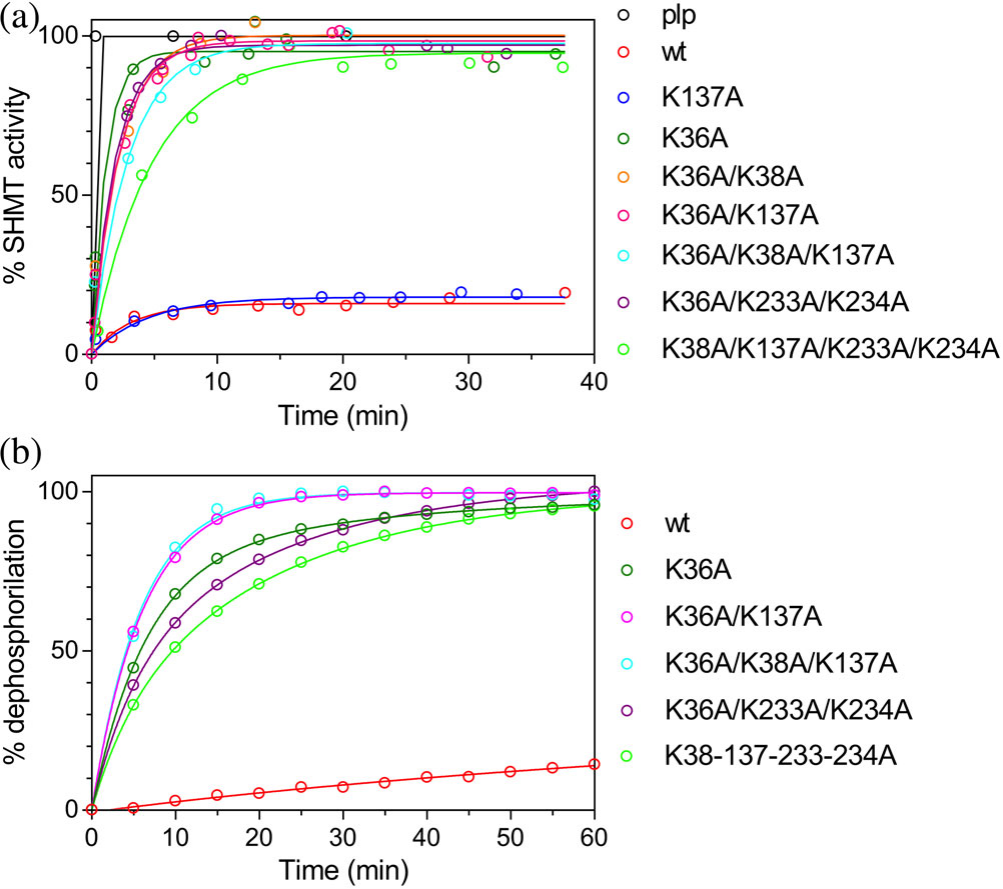
PLP transfer from YggS to *e*SHMT. (a) The activity of *e*SHMT (expressed as percentage of the total activity as a function of time) was measured as the initial velocity of the conversion of l‐serine and tetrahydrofolate to glycine and 5,10‐methylene tetrahydrofolate. The reactivation of apo‐*e*SHMT in the presence of 100 μM PLP (black line) was used as the 100% activity reference, assuming that in these conditions SHMT was completely in the holo‐form. Transfer kinetics were measured in the presence of the indicated YggS variants. (b) The kinetics of the PLP hydrolysis was measured in order to compare *k*_off_ of PLP dissociation from WT and variant forms of holo‐YggS, as explained in the text. Data are represented as percentage of hydrolyzed PLP, with respect to total protein‐bound PLP, as a function of time

We set up a novel in vitro discontinuous assay in which PLP transfer from holo‐YggS to the apo‐form of *e*SHMT could be followed (see Section 4 for details). In this assay, the increase in SHMT activity is measured as PLP dissociates from holo‐YggS and binds to apo‐SHMT, forming the catalytically active holo‐SHMT. A control experiment, in which YggS was replaced with a molar excess of free PLP, that rapidly and completely saturates apo‐SHMT, gave 100% SHMT activity as reference value. The binding kinetics of free PLP to either apo‐YggS or apo‐SHMT was measured by manually mixing 20 μM free PLP with 20 μM of protein, observing the formation of the internal aldimine at 420 nm. In both cases, PLP binding to *e*SHMT came to completion within the mixing time that lasted about 3 s (data not shown). Since YggS samples coming from standard purification procedures contained approximately 70–80% PLP with respect to protein monomers, we tried to obtain a fully saturated holo‐YggS to be used in the transfer experiments, by adding a slight excess of exogenous PLP with respect to the protein, and then eliminating all unbound PLP by SEC. However, a careful elimination of unbound PLP required a long SEC column, which returned an unsaturated YggS sample. On the other hand, if a shorter column was used, an apparently saturated sample was obtained; however, the PLP transfer kinetics obtained with this sample were clearly biphasic, showing a very rapid increase of holo‐SHMT formation clearly attributable to the presence of free PLP, which evidently had not been eliminated by SEC. Therefore, we employed a 70% saturated YggS sample as it came from the purification procedure, which ensured that virtually all PLP was bound to YggS. Transfer experiments, carried out at 37°C, used 20 μM holo‐YggS (28.6 μM total YggS concentration) and 20 μM apo‐SHMT, in 50 mM NaHEPES buffer at pH 7.6. An exponential PLP transfer kinetics was observed, with a rate constant of 0.14 ± 0.02 min^−1^, that reached the equilibrium in about 30 min (Figure 7a). At equilibrium, approximately 15% of PLP initially bound to YggS was transferred to SHMT.

### 2.11 Studies on the PLP transfer from YggS lysine variants to apo‐SHMT

PLP transfer kinetics were measured with all YggS lysine variants, except those that bind PLP very poorly (K36A/K38A/K233A/K234A, K36A/K137A/K233A/K234A, and K36A/K38A/K137A/K233A/K234A). While the single K137A variant showed the same transfer kinetics as that observed with WT YggS, variants that contained the K36A replacement (K36A, K36A/K38A, K36A/K137A, K36A/K38A/K137A, and K36A/K233A/K234A) showed a faster and much more extensive transfer of PLP to apo‐SHMT (Figure 7a). Interestingly, also the K38A/K137A/K233A/K234A variant showed PLP transfer properties similar to the latter variants. This is likely due to its reduced affinity for PLP when compared to the WT or the K137A variant.

It is notable that the percentage of transferred PLP seems to correlate with the *K*_D_ value of PLP binding of the lysine variants (Table 1). In order to assess whether the rate of PLP transfer depends on the apparent *k*_off_ value of PLP dissociation from holo‐YggS, the kinetics of PLP hydrolysis of WT and variant forms by PLP phosphatase was measured. Experiments were carried out in which 20 μM holo‐YggS samples were incubated with 0.5 μM PLP phosphatase, and the hydrolysis of PLP measured as the increase of absorbance at 318 nm, due to the formation of pyridoxal.^22^, ^23^ In these conditions, the small fraction of free PLP contained in the solvent, in equilibrium with protein‐bound PLP, is rapidly hydrolyzed by PLP phosphatase, promoting PLP dissociation from the protein and its subsequent hydrolysis. The observed rate of PLP hydrolysis therefore depends on the *k*_off_ of PLP dissociation from the protein. Figure 7b, in which PLP hydrolysis is reported as percentage of total protein‐bound PLP, shows that PLP hydrolysis takes place much more rapidly in the case of the YggS variants containing the K36A mutation, indicating that the rate of PLP transfer correlates with the *k*_off_ of PLP dissociation from the protein.

### 2.12 In vivo effects of lysine variants

**FIGURE 8:**
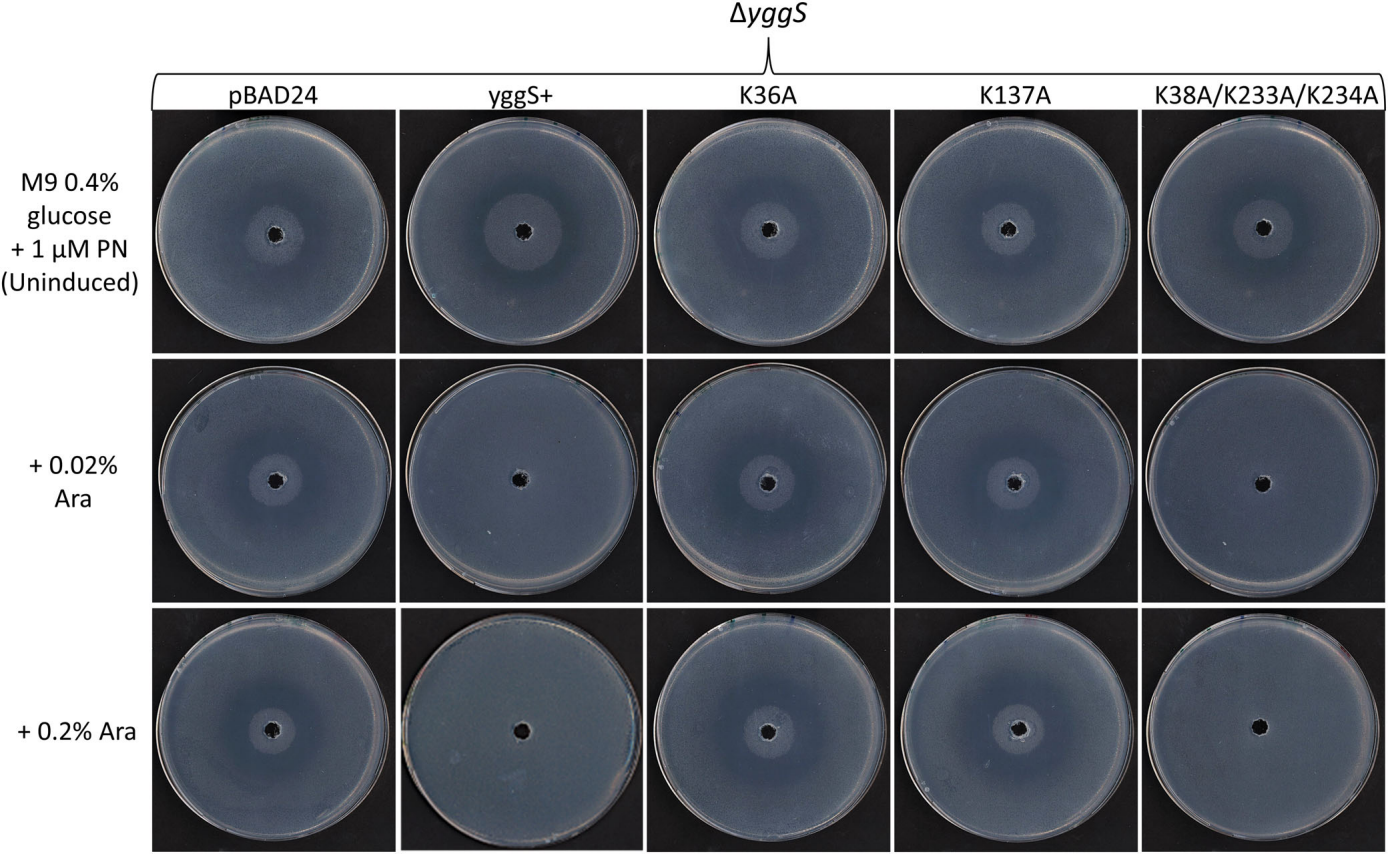
In vivo complementation of the 4‐deoxypyridoxine (4dPN) sensitivity phenotype by expression of YggS variants in trans. 4dPN sensitivity assays were carried out on M9 0.4% glucose minimal media supplemented with 1 μM PN (Amp 100 μg ml^−1^, +/− Arabinose [inducer]) as described in methods. Plated bacterial cells (OD_600_ = 0.006) were treated with 20 μl of 25 mM 4dPN in a central well, revealing a clear 4dPN sensitivity phenotype in *E*. *coli* Δ*yggS* that is complemented by expression of WT *yggS in trans*. Assays were repeated to investigate the ability of YggS lysine mutation variants to complement the 4dPN sensitivity phenotype, the figure shows that only the loss of K36 or K137 activity results in loss of complementation (all variant trials can be found in Figure S13). All plate sensitivity experiments were repeated at least three times and expression of each YggS variant was confirmed by western blot analysis (Figure S14)

An *E*. *coli yggS* deletion mutant (∆*yggS*) exhibits a concentration‐dependent sensitivity phenotype to the PN analog, 4dPN (Jill T. Babor and Valérie de Crécy‐Lagard, personal communication). This phenotype can be visualized by a distinct ring of growth inhibition on solid minimal media in the presence of PN and is complemented by expression of *YggS* in trans (Figure 8). In order to further investigate any physiological roles played by the lysine residues in vivo, Δ*yggS* strains harboring WT *YggS* and all various *YggS* lysine variant plasmids were constructed. 4dPN sensitivity assays were carried out to assess the ability of these YggS mutants to complement the 4dPN sensitivity phenotype of Δ*yggS*. Both K36A and K137A variant plasmids failed to complement the 4dPN sensitivity phenotype, while mutating the K38, K233, or K234 residues did not affect complementation (Figures 8 and S13). Further, analysis of variants with two, three, or four mutated lysine residues revealed that the absence of either K36 or K137 resulted in loss of YggS complementation, regardless of the presence of the remaining lysine residues (Figure S13). Western blot analysis confirmed that all mutant *yggS* genes analyzed were expressed to the same level as the WT (Figure S14).

## 3 DISCUSSION

Our general characterization of YggS showed some interesting features of this protein that had not been reported previously. One of these is the high affinity of YggS for PLP, indicated by a *K*_D_ of about 1 nM, which is much lower than that previously determined using other methods.^6^ PLP binding to apo‐YggS is very fast, as the binding equilibrium is fully reached in the manual mixing time, which is not longer than 3 s. On the other hand, PLP dissociates from YggS very slowly, as demonstrated by experiments in which holo‐YggS was incubated with PLP phosphatase. These observations indicate that PLP binding is characterized by a large *k*_on_ and a small *k*_off_, which is not really in favor of a role of PLP supplier for YggS. The large increase of *T*_m_ observed upon binding of PLP to apo‐YggS indicates an important stabilizing effect of this ligand as a result of relieving the repulsive forces among the positively charged lysine residues, such as those between K36 and K38. Based on the limited proteolysis experiments, it is tempting to suggest that this stabilizing effect might be accompanied by changes in either conformation or flexibility of the protein. However, we do point out that conformational changes are not evident from our crystallographic data of apo‐ and holo‐YggS. Notwithstanding, the crystallization process might have selected a single conformation of the protein. In fact, both the I4_1_22 and P4_1_2_1_2 cells show close or tightly packed YggS molecules in the crystal, which is likely restricting any conformational changes. A large stabilizing effect on apo‐YggS is also observed by increasing NaCl concentration, suggesting a neutralizing action of ions on protein charges. The stoichiometry of PLP binding to YggS is one molecule per protein monomer, with PLP covalently bound to Lys36 at the active site as shown by X‐ray crystallography (Figure 2). However, the addition of a large PLP concentration shows that a second PLP molecule binds non‐covalently to the protein (Figure 1), although with much lower affinity. This observation is confirmed by X‐ray crystallography data, which shows that the secondary PLP binding site is close to Lys89 residue (Figure 2). Since the *K*_D_ of this binding process is around 50 μM, we do not think that the binding of the second PLP molecule may have a particular function. Nevertheless, DSF experiments show that PLP binding at the secondary site further stabilizes the protein. Another interesting finding that had been missed in the previous studies^6^ is that PNP binds to apo‐YggS with relatively high affinity (*K*_D_ is about 30 nM), consistently with crystallographic studies that show PNP bound at the K36‐binding pocket in a similar fashion as PLP (Figure 2d). This observation suggests that in particular conditions in which PNP levels are increased, this vitamer may compete with PLP to bind to YggS.

A fundamental role in PLP binding to YggS is played by the Lys36 residue, which establishes an aldimine linkage with the aldehyde group of this vitamer. When Lys36 is replaced by an Ala residue, other Lys residues are able to bind PLP covalently, as demonstrated by the presence of a 420 nm absorbing band in the absorption spectrum of the K36A variant and by NaBH_4_ reduction experiments. The characterization of multiple lysine variants showed that K233/K234 and K38 may have a crucial role in binding PLP in the K36A variant. In fact, mass spectrometry analyses showed that K38 binds PLP in the K36A variant. However, PLP was not found to be covalently bound to any Lys residue in the crystal structures of the protein containing the K36A mutation. This observation is not in contrast with the spectroscopic data acquired with these protein forms in solution, which indicate the presence of a Schiff base linkage. Evidently, the crystallization of the protein selected a conformation in which the Schiff base linkage with alternative lysine residues is unfavorable. The above‐mentioned restrictions on protein flexibility imposed by protein interactions in the crystal, and the PLP binding site that is exposed to a large solvent channel, are expected to affect the reversible equilibrium of internal aldimine formation.

All lysine residues taken into consideration play a role in protein stability, since the apo‐form of all lysine variants show an increased *T*_m_ with respect to WT YggS. In the case of the K36A variant, binding of PLP does not elicit an increase in *T*_m_, suggesting that the protein structure is already in a form that resembles that of the WT holo‐form. This hypothesis is supported by limited proteolysis experiments, which show how the rate of protein cleavage in K36A apo‐form is similar to WT holo‐form, and how the addition of PLP to K36A apo‐form does not affect the rate of protein cleavage. On the other hand, the apo‐form of K38A/K137A/K233A/K234A variant, which has Lys36 to bind PLP covalently but lacks the surrounding Lys residues, also shows increased *T*_m_ with respect to apo‐WT, and a limited proteolysis pattern very similar to that of the K36A variant. These observations suggest that the energetic frustration^34^ due to the presence of K36 at the active site of apo‐YggS is determined by unfavorable electrostatic interactions of this residue with a sort of a “lysine cluster” represented by K38, K137, and K233/K234, which are quite close to K36 and localized at the entrance of the protein active site (Figure S10). The observed stabilizing effect of NaCl is in agreement with this hypothesis. Although the C‐terminal end of YggS is not visible in the electron density map of all crystallized YggS variants, it may be envisaged that K233 and K234 residues are localized close to the PLP binding site, and therefore in proximity to the other Lys residues of the cluster (Figure S10). In fact, the effect of K38A and K137A replacements on the conformation or flexibility of the C‐terminal end of the protein is visible from the limited proteolysis experiments that show how these mutations expose Arg229 to tryptic cleavage. Lysine residues K38 and K233‐K234, that bind PLP as an internal aldimine when K36 is absent, are not invariant in a multiple alignment of YggS from eight different eukaryotic and prokaryotic sources (Figure S6); however, the “lysine cluster,” considered more broadly (including also K65, K89, and K137) as the positively charged residues that surround the active site, shows a good degree of conservation. We believe that the covalent linkage of PLP by K38 and K233‐K234 observed when K36 is replaced by an alanine residue may only be an accidental consequence of the presence of these lysine residues at the active site entrance, whereas the “lysine cluster” as a whole may actually play an important structural and functional role in YggS.

The fundamental role of K36 and K137 residues in the function of YggS is evident from in vivo experiments. The K36A variant was unable to complement 4dPN sensitivity, suggesting that the alternative lysine residues, although able to bind PLP covalently, are unable to replace the functional role of Lys36. Another important observation is that the K137A variant, although has no effects on PLP binding and transfer properties of the protein, has a drastic effect on the cellular function of YggS, suggesting that the K137A residue must play a role in the function of YggS that is not directly connected to PLP binding and transfer. Alternatively, since K137 is located on a large loop exposed to solvent, at the active site entrance of the protein, this residue might be involved in interactions with other macromolecules. Another interesting finding, coming from site‐directed mutagenesis and spectroscopic studies, is that YggS apparently binds PLP covalently to K233A/K234A when Lys36 is mutated to Ala, although this suggestion is not supported by X‐ray crystallography and mass spectrometry, the former due to high flexibility of the C‐terminal loop not allowing amino acid residues to be resolved beyond R229 in the crystal structure.

The dissociation constant (4.8 μM) determined in SPR experiments suggests that an interaction between *e*SHMT and YggS occurs, although weak and likely transient. A large number of biological processes involve weak or transient protein–protein interactions (with dissociation constants from 10^−3^ to 10^−6^ M) that are essential for their activities.^35^, ^36^ It is not clear whether this interaction may be at the basis of the observed PLP transfer process. The experiments carried out in this work cannot discriminate between the two possible mechanisms of PLP transfer: a direct channeling or a simple dissociation of PLP from YggS followed by its association with SHMT. These two transfer possibilities will be analyzed in future investigations. PLP transfer experiments in which holo‐YggS was a donor and apo‐*e*SHMT an acceptor show that YggS is actually able to transfer PLP, however, the observed slow transfer kinetics are not in favor of a role of YggS as a PLP donor. It should also be considered that the large concentrations of YggS and *e*SHMT used in our PLP transfer experiments (20 μM each) are undoubtedly far larger than the cellular concentrations of these proteins. Therefore, it is expected that PLP transfer kinetics would be much slower in the cellular conditions. The PLP transfer equilibrium point, which corresponds to about 15% of PLP transferred from WT holo‐YggS to apo‐*e*SHMT, is in agreement with the fivefold larger *K*_D_ for PLP measured with the latter enzyme (about 5 nM). Accordingly, the transfer equilibrium point of YggS lysine variants (which corresponds to 90% of transferred PLP) correlates with the higher *K*_D_ for PLP of these protein forms. A physiological role of YggS as a “PLP sponge” in the cell, which sequesters free PLP from the environment as this is produced and contributes to PLP homeostasis, would only be possible if YggS concentration in the cell were large enough to actually absorb large amounts of PLP; moreover, in order to redistribute PLP, YggS should also have a lower affinity for PLP than PLP‐dependent apoenzymes. However, studies on the cellular concentration of *E*. *coli* proteins indicated that YggS is not an abundant protein, not even more abundant than SHMT for instance.^37^, ^38^ In summary, based on our results, YggS might not be suitable to play the proposed role of PLP‐carrier/distributor, and not even that of PLP reservoir in the cell. After all, a Δ*yggS E*. *coli* strain grows normally on a minimal medium containing only salts and glucose,^6^ and this observation is not compatible with such hypothetic, fundamental functions.

Some features of YggS, such as the high affinity for PLP and the possible change in conformation and/or flexibility induced by PLP binding, together with the striking results obtained in the in vivo experiments, point toward a role of YggS as a component of an unknown regulatory pathway. The concentration of free PLP in the cell is low since most of this cofactor is bound to proteins.^4^ Therefore, a signaling protein whose role was to sense PLP levels should have a high affinity for this cofactor. The binding of PLP to YggS, and the consequent changes in protein conformation or flexibility, may regulate its interactions with other cellular components involved in vitamin B_6_ homeostasis.

## 4 MATERIALS AND METHODS

### 4.1 Growth conditions and media

Bacteria were grown in Luria‐Bertani (LB; Tryptone 10 g L^− 1^, Yeast extract 5 g L^− 1^, NaCl 10 g L^− 1^) or M9 minimal medium (Cold Spring Harbor Protocols, Cold Spring Harbor, NY) at 37°C, unless otherwise specified. Growth media were solidified with agar (16 g L^− 1^; Fisher Scientific) for the preparation of plates. 4dPN toxicity assays were performed on thin (20 ml) M9 minimal medium plates containing 0.4% glucose (carbon source), ampicillin (100 μg ml^−1^), 1 μM PN, and 0.02% or 0.2% arabinose when appropriate.

### 4.2 Expression and purification of WT and variant YggS forms

The coding sequence of *yggS* was amplified by PCR using the primers pETyggS_for/pETyggS_rev (Table S1) and genomic DNA from *E*. *coli* BW25113 as template. The amplicon was inserted into a pET28b(+) vector between NcoI and XhoI restriction sites to obtain the pET28*yggS* plasmid (Table S2), which allows the expression of YggS with a His‐tag at the C‐terminal end. This plasmid was used as template for the production of single variants (Table S2). Multiple variants were produced by sequentially adding single mutations on the same template plasmid. Site‐directed mutagenesis was carried out using a standard protocol described in the Quick‐Change kit from Stratagene (La Jolla, CA). For each mutation, two complementary oligonucleotide primers, synthesized by Metabion International AG (Steinkirchen, Germany) and listed in Table S1, were used. The WT *yggS* coding region was also inserted into the pET28b(+) vector between NdeI and EcoRI restriction sites to obtain the expression of YggS with a His‐tag at the N‐terminal end (Table S2). This plasmid was used to produce the ΔK233/K234 variant. *E*. *coli* DH5α cells were used to amplify all the plasmids, whereas *E*. *coli* HMS174(DE3) was used for the expression of all the WT and variant YggS forms. All the mutations were confirmed by sequence analysis (Microsynth AG, Balgach, Switzerland).

For the purification of all YggS variants, an overnight culture (40 ml) of *E*. *coli* HMS174(DE3) cells transformed with appropriate plasmids was used to inoculate 4 L of LB medium. Bacteria were allowed to grow for approximately 3 hr at 37°C (until their OD600 reached to ~0.6), then the growing temperature was lowered to 28°C and the expression of YggS induced with 0.2 mM isopropyl thio‐β‐d‐galactoside (IPTG). Bacteria were harvested after 18 hr and suspended in 150 ml of 20 mM KPi pH 7.3 buffer, containing 150 mM NaCl (buffer A) and 2 mg/ml lysozyme. Cell lysis was carried out with three freezing/defrosting cycles and by sonication on ice (3‐min in short 20‐s pulses with 20‐s intervals). Lysate was centrifuged at 12,000 *g* for 30 min and the pellet was discarded. The supernatant was precipitated by the addition of ammonium sulfate to 25% saturation and then to 60% saturation, and centrifuged for 15 min at 13,000 rpm. The pellet was collected and resuspended in buffer A and loaded onto a 10 ml Ni‐NTA Superflow (QIAGEN, Germantown, MD) column, previously equilibrated with buffer A, the column was washed with 50 ml of buffer A and eluted with the same buffer and a linear 0–300 mM imidazole gradient (the buffer containing imidazole was adjusted to pH 7.3 using HCl). Fractions containing YggS were collected and dialyzed against 50 mM NaHEPES buffer pH 7.5 containing 2.5 mM 2‐mercaptoethanol. The characterization of purified YggS with the His‐tag at either the N‐terminal or the C‐terminal end showed that these forms of the protein have similar chemico‐physical, PLP binding, and PLP transfer properties.

The wild‐type and variant apo‐forms of YggS were prepared using l‐cysteine.^39^ About 15 mg of YggS was incubated with 100 mM cysteine. Cysteine rapidly reacted with PLP forming a thiazolidine complex, absorbing at 330 nm. Subsequently, apo‐protein, thiazolidine complex, and cysteine in excess was separated on phenylsepharose column (5 ml), equilibrated with 50 mM NaHEPES, pH 7.6, containing 25% ammonium sulfate and 50 mM l‐cysteine. The column was washed with one volume of the equilibration buffer and then with three volumes of 50 mM NaHEPES, pH 7.6, containing 25% ammonium sulfate. Thiazolidine is washed away with this last buffer (checked by measuring the absorption spectrum of the eluate). The apo‐YggS was eluted with 50 mM NaHEPES, pH 7.6.

### 4.3 Spectroscopic measurements

All spectroscopic measurements were carried out at 20°C in 20 mM NaHEPES pH 7.5. UV–visible spectra were recorded with a Hewlett‐Packard 8453 diode‐array spectrophotometer. Far‐UV (190–250 nm) and Near‐UV–visible (250–500 nm) CD spectra were measured with a Jasco 710 spectropolarimeter equipped with a DP 520 processor using 0.1‐cm (far‐UV) or 1‐cm path length quartz cuvettes and results were expressed as ellipticity [Θ]. Absorption spectra of WT and variant forms were deconvoluted into their component bands using Metzler's band‐shape analysis method.^22^

### 4.4 Analysis of PLP binding equilibrium

Analyses took advantage of YggS intrinsic fluorescence decrease observed upon binding of PLP. Dissociation constants were calculated from saturation curves obtained measuring the protein fluorescence emission intensity as a function of increasing PLP concentration. Fluorescence emission measurements were carried out at 25°C, in 50 mM NaHEPES buffer at pH 7.6 with a FluoroMax‐3 Jobin Yvon Horiba spectrofluorometer, using a 1‐cm path length quartz cuvette. All analyzed YggS forms were used at a final subunit concentration of 0.1 μM. Fluorescence emission spectra were recorded from 310 to 450 nm upon excitation at 280 nm. Excitation and emission slits were set at 3 and 5 nm, respectively. Emission fluorescence values between 330 and 340 nm were averaged and analyzed using the following quadratic equation to obtain *K*_D_ value:

(1) $$F_{\mathrm{rel}}=\begin{array}{l} \left(F_{\inf}-F_{0}\right)\times \frac{\frac{\left[\mathrm{PLP}\right]+\text{[YggS]}+K_{D}-\sqrt{{\left(\left[\mathrm{PLP}\right]+\text{[YggS]}+K_{D}\right)}^{2}-4\text{[YggS]}\left[\mathrm{PLP}\right]}}{2}}{\text{[YggS]}}+\;F_{0} \end{array}$$

### 4.5 Differential scanning fluorimetry assays

Differential scanning fluorimetry (DSF) assays were performed on a Real Time PCR Instrument (CFX Connect Real Time PCR system, Bio‐Rad, Hercules, CA). In a typical experiment, 2 μM WT and variant apo‐YggS forms in 50 mM NaHEPES, pH 7.5, 50 mM NaCl, and Sypro Orange (5x, Thermo Scientific) were mixed with various ligands (total volume of 25 μl) in a 96‐well PCR plate. Fluorescence was measured from 25°C to 95°C in 0.4°C/30 s steps (excitation 450–490 nm; detection 560–580 nm). All samples were run in triplicate. Denaturation profiles were analyzed as described in Nardella et al.^40^

### 4.6 Gel filtration analyses

Gel filtration of YggS wild‐type and variant forms were performed on a Superdex 200 10/300 GL column (GE Healthcare, Little Chalfont, UK) at room temperature and at a flow rate of 0.5 ml/min in 50 mM NaHEPES pH 7.5, containing 150 mM NaCl and 2.5 mM β‐mercaptoethanol. Elution profiles were obtained from absorbance at 280 nm. Calibration was performed with aldolase (158 kDa), conalbumin (75 kDa), ovalbumin (44 kDa), carbonic anidrase (29 kDa), and RNase A (17.7 kDa).

### 4.7 Analytical ultracentrifuge

A sedimentation velocity (SV) experiment using absorbance and interference detectors was carried out using a Proteome Lab XL‐I analytical ultracentrifuge (AUC; Beckman Coulter) as per Aljahdali et al.^41^ YggS protein (1.5 mg) was loaded into AUC cell assemblies in PBS, pH 7.4 and centrifuged at 40,000 rpm, for 9 hr at 20°C. The resulting SV data was analyzed using the continuous c(s) module in SEDFIT version 16.36 as described in Schuck et al.^42^

### 4.8 Peptide analysis by mass spectrometry

Analyses were performed by the Mass Spectrometry Incubator facility of the Virginia Tech Department of Biochemistry in Blacksburg, Virginia. An aliquot of the YggS variant (K36A) containing approximately 100 μg protein was precipitated by the addition of LC–MS grade methanol (1 ml) and storage at −80°C overnight. Precipitated protein was collected by centrifugation at 7°C for 15 min (13,000×*g*). Protein was solubilized in 100 mM HEPES, pH 7.8 (200 μl). PLP‐protein reduction was performed by the addition of 1 M NaBH_4_ (10 μl), and the sample incubated in the dark at 37°C for 1 hr. Protein was again precipitated and the pellet collected as described above. The protein pellet was then dissolved in S‐Trap lysis buffer (100 μl), which contained 5% SDS, 50 mM triethylammonium bicarbonate, pH 8.5 (TEAB). Disulfide bonds were reduced by the addition of 4.5 mM dithiothreitol (DTT) and then alkylated by the addition of 10 mM iodoacetamide (IAA). DTT was then added at 10 mM to quench any remaining IAA. The sample was acidified by the addition of 1.2% (v/v) *o*‐phosphoric acid and protein was again precipitated as above. The precipitated protein was loaded onto a microspin S‐Trap column in aliquots of 120 μl by centrifugation at room temperature (1 min; 1,000×*g*). The precipitated protein on the column was washed with LC–MS grade methanol (120 μl, 4×) and then digested using MS grade Pierce™ Glu‐C protease/ Trypsin (10 μg) in 50 mM TEAB (25 μl) at 37°C overnight. Peptides were collected by washing the S‐Trap serially with mixtures of Solvent A (LC–MS acetonitrile) and Solvent B (80:20 LC–MS acetonitrile: water, 0.1% (v/v) formic acid) in the following sequence: 2:98 (A:B, 25 μl), 1:1 (A:B, 25 μl), and 100% B (25 μl). Samples were centrifuged at room temperature for 1 min at 1,000×*g* after each addition. Acetonitrile was removed by centrifugal vacuum concentration.

An aliquot containing approximately 0.2 μg was then analyzed via LC–MS/MS as described below. A portion of the remaining peptide sample (45 μg in 17 μl) was mixed with 10X CutSmart Buffer (2 μl, New England Biolabs) and Quick CIP phosphatase (1 μl, New England Biolabs) and incubated at 37°C for 30 min. The Quick CIP phosphatase was then inactivated by incubation at 80°C for 2 min. The sample was acidified by the addition of Solvent A (180 μl) and desalted using a C_18_ SPE tip. Acetonitrile was removed by centrifugal vacuum concentration and an aliquot containing approximately 0.2 μg of the dephosphorylated sample was then analyzed via LC–MS/MS as described below.

Samples were first loaded onto a precolumn (Acclaim PepMap 100 [ThermoFisher], 100 μm × 2 cm) after which flow was diverted to an analytical column (50 cm μPAC; PharmaFluidics). The UPLC/autosampler utilized was an Easy‐nLC 1200 (ThermoFisher). Flow rate was maintained at 150 nl/min and peptides were eluted utilizing a 2–45% gradient of Solvent B in Solvent A over 88 min. The mass spectrometer utilized was an Orbitrap Fusion Lumos Tribid™ from ThermoFisher. Spray voltage on the μPAC compatible Easy‐Spray emitter (PharmaFluidics) was 1.3 kV, the ion transfer tube was maintained at 275°C, the RF lens was set to 30%, and the default charge state was set to 3.

MS data for the *m/z* range of 400–1,500 was collected using the orbitrap at 120,000 resolution in positive profile mode with an AGC target of 4.0e5 and a maximum injection time of 50 ms. Peaks were filtered for MS/MS analysis based on having isotopic peak distribution expected of a peptide with an intensity above 2.0e4 and a charge state of 2–5. Peaks were excluded dynamically for 15 s after one scan with the MS/MS set to be collected at 45% of a chromatographic peak width with an expected peak width (FWHM) of 15 s. MS/MS data starting at *m*/*z* of 150 was collected using the orbitrap at 15,000 resolution in positive centroid mode with an AGC target of 1.0e5 and a maximum injection time of 200 ms. Activation type was HCD stepped from 27 to 33.

Proteome Discoverer v. 2.5 (ThermoFisher) was used to generate peak lists from the LC–MS/MS data. Then initial error‐tolerant searches for each run using Mascot v. 2.7 (Matrix Science), to find any known modifications, and Byonic v. 3.11 (Protein Metrics), to find unexpected mass shifts not related to any known modifications, were conducted using a database containing common laboratory protein contaminants and the reference proteome database for *Escherichia coli* (strain K12) downloaded August 21, 2020, and appended with an entry representing the PLPHP variant (K36A) using UniProt ID P30074 as the header to distinguish it from the wild‐type protein. The results of these searches identified possible modifications present and ensured protease specificity. Second specific searches were then conducted using Mascot and only the modifications of interest identified in the first series of error‐tolerant searches.

### 4.9 Limited proteolysis experiments and mass spectrometry measurements

Protein samples (6.5 μM) of apo‐YggS forms with and without 20 μM PLP were incubated with trypsin from bovine pancreas (0.005 mg/ml; Sigma‐Aldrich, Darmstadt, Germany) at 20°C in 50 mM NaHEPES, pH 7.6, containing 2.5 mM β‐mercaptoethanol. At specific time intervals, the proteolytic reaction was stopped by the addition of SDS, and samples were boiled. Samples were then analyzed by SDS‐PAGE and revealed with Comassie Brilliant Blue R‐250. Interesting bands were proteolyzed in‐gel by trypsin (Trypsin Gold, Promega). Each peptide mixture was analyzed on a MALDI‐ToF/ToF (ultrafleXtreme, Bruker, Bremen, Germany) mass spectrometer, equipped with a smartbeam‐II laser. The m/z values of any spectra were compared with the theoretical mass list of expected YggS tryptic peptides and validated by tandem mass experiments, in LIFT mode.

### 4.10 Surface Plasmon resonance assay

Surface Plasmon resonance (SPR) experiments were carried out using a SensiQ Pioneer system, essentially as in Genovese et al. and Illari et al.^43^, ^44^ The sensor chip (COOH5) was activated chemically by a 35 μl injection of a 1:1 mixture of N‐ethyl‐N′‐(3‐diethylaminopropyl) carbodiimide (200 mM) and N‐hydroxysuccinimide (50 mM) at a flow rate of 5 μl/min. The ligand, that is, apoSHMT, was immobilized on activated sensor chips via amine coupling. The immobilizations were carried out in 20 mM sodium acetate at pH 4.0; the remaining unreacted groups were blocked by injecting 1 M ethanolamine hydrochloride (100 μl). The immobilization level of apoSHMT was 100 RU.

The analyte (wild‐type YggS) was dissolved at a concentration of 120 μM in PBS containing 0.005% surfactant P20 (PBSP buffer) and automatically further diluted in PBSP buffer (running buffer) and injected on the sensor chip at the following concentrations: 0.24, 0.72, 2.2, 6.7, 20, 60, and 180 μM at a constant flow (30 μl/min). The increase in Resonance Units (RU) relative to baseline indicates complex formation; the plateau region represents the steady‐state phase of the interaction (RUeq), whereas the decrease in RU after 240 s represents dissociation of analytes from immobilized SHMT after injection of PSBP buffer. Regeneration procedures are based on two long (1,000 s and 500 s) injections of buffer, separated by a brief (5 s) injection of 10 mM NaOH. The sensorgrams were analyzed using the SensiQ Qdat program, and Scatchard analysis.

### 4.11 PLP transfer assays

Recombinant *E*. *coli* SHMT was expressed and purified as described by Contestabile et al.^45^ The apo‐*e*SHMT form used in this experiment was prepared using the same procedure described for the production of apo‐YggS (see above).

WT Holo‐YggS (20 μM with respect to PLP) was mixed with 20 μM apo‐*e*SHMT in 50 mM NaHEPES buffer at pH 7.6. At time intervals, aliquots of this mixture were diluted 1:400 into a spectrophotometer cuvette containing all ingredients to assay the SHMT activity (10 mM l‐Serine, 50 μM tetrahydrofolate, 250 μM NADP^+^, 5 μM 5,10‐methylene tetrahydrofolate dehydrogenase in the same NaHEPES buffer, at 37°C).^46^ Dilution stopped the PLP transfer process but did not affect the amount of PLP transferred to SHMT, as demonstrated by control experiments, showing that PLP does not dissociate from holo‐SHMT when this is diluted 1:400 into the spectrophotometer cuvette.

### 4.12 Structure determination

His‐tagged purified YggS protein was dialyzed against 50 mM Potassium Phosphate/or Tris–HCl pH range 7.2–7.5 containing 150 mM NaCl, and concentrated to 30–50 mg/ml. Crystallization experiments were carried out using Crystal Gryphon robot (Art Robbins Instruments) at 20°C. A wide range of commercially available crystallization conditions were screened. Then, 58 μl reservoir solution and 400 nl crystallization drops were dispensed on 96‐well INTELLI‐PLATES by Gryphon Dispenser. Attempts to improve the quality and size of crystals using sitting drop vapor diffusion method was performed up to microliter range in 24‐well VDX crystallization plates (Hampton Research). Suitable crystals of wild‐type YggS, as well as several mutants of YggS with or without PLP or PNP were obtained in 2 weeks with several different precipitants, and further refinement of conditions resulted in diffracting crystals mainly from sulfate/or phosphate salts as precipitants. Crystallization conditions of the structures reported here are presented in Table S3.

Prior to data collection, the crystals were cry‐protected by washing in 2.5 μl corresponding crystallization solutions containing 15–25% glycerol. X‐ray data sets of the crystals were obtained at 100 K using Rigaku MicroMax™ 007HF X‐ray Generator, Eiger R 4M Detector, and Oxford Cobra Cryo‐system (The Woodlands, TX). The diffraction data were processed using CrysAlis PRO program (Rigaku Oxford Diffraction Ltd. Yarnton, Oxfordshire, England). The crystal structures were solved by a molecular replacement method with the Phenix program,^47^ using the *E*. *coli* K‐12 YggS crystal structure (PDB ID 1W8G) as a search model. The structure was refined using Phenix, while model building and correction were carried out using COOT.^47^, ^48^, ^49^ The atomic coordinates and structure factors are deposited in the RCSB Protein Data Bank and the entries are shown in Table 3 along with other crystallographic parameters.

### 4.13 Plasmids for in vivo 4dPN sensitivity assays

Wild‐type *ygg*S was amplified by PCR with primers DH596 and DH598 from *E*. *coli* BW25113 and cloned in pBAD24 using restriction sites *Sph*I and *Nco*I to give pBY291.3. The pBY291.3 plasmid and its derivatives were then used as templates with the NEB Q5 Site‐Directed Mutagenesis Kit and the primer pairs indicated in Table S4 to introduce site‐directed residue mutant constructs. All constructs were verified by sequencing and transformed into *E*. *coli* wild‐type (BW25113) and Δ*yggS* (VDC6594) electrocompetent cells for use in physiological studies. Plasmid transformations were performed following standard procedures and protein expression from each construct was confirmed by SDS‐PAGE and western blot analysis.

### 4.14 4dPN sensitivity assays

Three isolated colonies of each strain were inoculated into 5 ml LB Amp 200 μg ml^− 1^ and incubated at 37°C and 200 rpm overnight. Overnight cultures were diluted 1:500 into 5 ml fresh LB Amp 200 μg ml^− 1^ ± 0.02% or 0.2% arabinose and grown at 37°C, shaking. Once cultures reached midlog growth (OD_600_ of 0.4–0.7) samples were taken from each culture for western blot analysis (below) then cells were again incubated until stationary growth was reached (~6 hr). Cells were harvested by centrifugation then washed twice with and resuspended in 1x PBS. Optical density of washed cells was measured at 600 nm in 1 ml cuvettes (Fisher Scientific) using a BioSpec‐mini DNA/RNA/Protein analyzer (Shimadzu Biotech) and each strain was normalized to an OD_600_ of 0.006 in 3 ml 1x PBS. Cells were then plated on minimal media plates by pouring the normalized cell dilutions onto the plate, rotating to ensure complete coverage, removing excess culture by pipetting, and letting the plates dry for 5–10 min on benchtop. Wells were made in the center of each plate using the large end of sterile 200 μl pipette tips and 20 μl of 25 mM 4dPN was placed at the well center. Plates were incubated upright at 37°C for 24–36 hr and imaged using a Cannon Scan 5600F scanner. All experiments were repeated three times independently.

### 4.15 Western blot to confirm in vivo protein expression of plasmid constructs during 4dPN sensitivity assays

Western blot samples of experimental strains were obtained at mid‐log growth phase (OD_600_ of 0.4–0.7) by collecting cells from 1 ml of culture media by centrifugation and resuspending in 2× Laemmli sample buffer (2× LSB: 4% SDS; 10% 2‐mercaptoethanol; 20% glycerol; 0.004% bromophenol; 0.125 M Tris HCl; pH 6.8) to normalize: if OD_600_ = 0.45, resuspend in 45 μl 2× LSB. Samples were boiled for 5 min at 100°C then 10 μl of each was loaded into 12% SDS‐polyacrylamide gels. Gel electrophoresis was performed at 30 mA (constant) until loading dye reached the end of the gel (~1 hr) in SDS buffer. Proteins were transferred from the gels to activated PVDF membranes using the BioRad TransBlot‐SD semi‐dry transfer cell at 10 V (constant) for 15 min. Membranes were incubated in blocking buffer (5% nonfat milk PBS‐T for 2 hr, rocking at room temperature), primary antibody (1:5000 in PBS‐T solution, polyclonal antibodies generated in New Zealand Rabbit against purified WT‐YggS, rocking at room temperature for 1 hr), and secondary antibody (1:5000 Goat α‐Rabbit (HRP conjugate) polyclonal secondary antibody (0.5 mg/ml, Thermo Scientific) PBS‐T solution for 45 min, rocking at room temperate) solutions, rinsing well with PBS‐T between each incubation. HRP secondary antibody signal was activated via incubation in fresh 1:1 HRP substrate (stable peroxide solution:luminol/enhancer solution; Thermo Scientific) for ~3 min, then imaged using Invitrogen iBright FL1000 gel imager, chemiluminescence detection.

## AUTHOR CONTRIBUTIONS

**Angela Tramonti:** Conceptualization (equal); data curation (equal); formal analysis (equal); funding acquisition (equal); investigation (equal); methodology (equal); validation (equal); visualization (equal); writing – original draft (equal); writing – review and editing (equal). **Mohini S. Ghatge:** Conceptualization (equal); data curation (equal); formal analysis (equal); investigation (equal); methodology (equal); validation (equal); visualization (equal); writing – original draft (equal); writing – review and editing (equal). **Jill T. Babor:** Data curation (equal); formal analysis (equal); investigation (equal); methodology (equal); validation (equal); visualization (equal). **Faik N. Musayev:** Data curation (equal); formal analysis (equal); investigation (equal); methodology (equal); visualization (equal). **Martino L. di Salvo:** Investigation (equal); methodology (equal). **Anna Barile:** Investigation (equal); methodology (equal). **Gianni Colotti:** Investigation (equal); methodology (equal). **Alessandra Giorgi:** Investigation (equal); methodology (equal). **Steven D. Paredes:** Investigation (equal); methodology (equal). **Akua K. Donkor:** Investigation (equal); methodology (equal). **Mohammed H. Al Mughram:** Investigation (equal); methodology (equal). **Valerie de Crécy‐Lagard:** Conceptualization (equal); formal analysis (equal); funding acquisition (equal); project administration (equal); resources (equal); supervision (equal); validation (equal); writing – original draft (equal); writing – review and editing (equal). **Martin K. Safo:** Conceptualization (equal); data curation (equal); formal analysis (equal); funding acquisition (equal); investigation (equal); methodology (equal); resources (equal); supervision (equal); validation (equal); visualization (equal); writing – original draft (equal); writing – review and editing (equal). **Roberto Contestabile:** Conceptualization (equal); funding acquisition (equal); resources (equal); supervision (equal); writing – original draft (equal); writing – review and editing (equal).

## CONFLICT OF INTEREST

The authors declare that they have no conflicts of interest with the contents of this article.

## Supporting information


**Table S1.** Oligonucleotide primers used in this study
**Table S2.** Plasmids used for the expression of YggS variants
**Table S3.** YggS crystallization conditions
**Table S4.** Plasmid constructs for in vivo 4dPN sensitivity assays of YggS‐Lysine mutants
**Figure S1.** Time course of Schiff base formation and titration of PLP binding through Schiff base formation
**Figure S2.** PL and PNP binding equilibria
**Figure S3.** DSF measurements of apo‐YggS in the presence of different concentrations of PLP, PL and PNP
**Figure S4.** Size exclusion chromatography and AUC analyses of WT YggS
**Figure S5.** Superposition of holo‐YggS with 1W8G, 5NM8, and 1CT5
**Figure S6.** Multiple sequence alignment of YggS from eukaryotic and prokaryotic sources
**Figure S7.** Deconvolution of absorption spectra of YggS forms
**Figure S8.** Absorption spectra of protein samples and small molecular weight samples after NaBH_4_ treatment
**Figure S9.** DSF measurements of apo‐YggS variants in the presence of different concentrations of PLP
**Figure S10.** Cartoon representation of WT holo‐YggS crystal structure solved and described in this work
**Figure S11.** Far‐UV CD spectra of WT and variant YggS forms
**Figure S12.** SPR analyses of YggS‐SHMT interactions
**Figure S13.** In vivo complementation of the 4‐deoxypyridoxine (4dPN) sensitivity phenotype by expression of all YggS lysine variants *in trans*
**Figure S14.** Western blots for verification of functional protein expression of YggS forms
