# Supplementary material for: Characterization of the Escherichia coli pyridoxal 5′‐phosphate homeostasis protein (YggS): Role of lysine residues in PLP binding and protein stability
Source: Protein Sci. 2022 Oct 26;31(11):e4471. doi: 10.1002/pro.4471 (PMC9601805; doi:10.1002/pro.4471)
Supplement: Supplementary file 1 — Table S1. Oligonucleotide primers used in this study Table S2. Plasmids used for the expression of YggS variants Table S3. YggS crystallization conditions Table S4. Plasmid constructs for in vivo 4dPN sensitivity assays of YggS‐Lysine mutants Figure S1. Time course of Schiff base formation and titration of PLP binding through Schiff base formation Figure S2. PL and PNP binding equilibria Figure S3. DSF measurements of apo‐YggS in the presence of different concentrations of PLP, PL and PNP Figure S4. Size exclusion chromatography and AUC analyses of WT YggS Figure S5. Superposition of holo‐YggS with 1W8G, 5NM8, and 1CT5 Figure S6. Multiple sequence alignment of YggS from eukaryotic and prokaryotic sources Figure S7. Deconvolution of absorption spectra of YggS forms Figure S8. Absorption spectra of protein samples and small molecular weight samples after NaBH4 treatment Figure S9. DSF measurements of apo‐YggS variants in the presence of different concentrations of PLP Figure S10. Cartoon representation of WT holo‐YggS crystal structure solved and described in this work Figure S11. Far‐UV CD spectra of WT and variant YggS forms Figure S12. SPR analyses of YggS‐SHMT interactions Figure S13. In vivo complementation of the 4‐deoxypyridoxine (4dPN) sensitivity phenotype by expression of all YggS lysine variants in trans Figure S14. Western blots for verification of functional protein expression of YggS forms [file PRO-31-e4471-s001.docx]

**Table S1.** Oligonucleotide primers used in this study. Mutated codons are shown in red.

| Primer | Sequence (5’-3’) |
| --- | --- |
| pETyggS_for | GGCCATGGACGATATTGCGCATAACCTG |
| pETyggS_rev | GGCTCGAGTTTTTTAGAGTAATCACGCGCACC |
| pETyggS2_for | CGGCATATGAACGATATTGCGCATAACCTG |
| pETyggS2_rev | CGCGAATTCTTATTTTTTAGAGTAATCACGCGCAC |
| YggS K36A_for | CAGTCAGTGCAACAAAACCTGCGAGCG |
| YggS K36A_rev | GGTTTTGTTGCACTGACTGCAAGCAGC |
| YggS K36A/K38A_for | TGCAACAGCGCCTGCGAGCGCCATC |
| YggS K36A/K38A_rev | CTCGCAGGCGCTGTTGCACTGACTG |
| YggS K38A_for | TAAAACAGCGCCTGCGAGCGCCATC |
| YggS K38A_rev | CTCGCAGGCGCTGTTTTACTGACTG |
| YggS K137A_for | AGTGATGCAAACAGTAAGTCCGG |
| YggS K137A_rev | CCCGGACGCACTGTTTTCATC |
| YggS K233A/K234A_for | GCGTGATTACTCTGCAGCACTCGAGCACCACC |
| YggS K233A/K234A_rev | CGCACTAATGAGACGTCGTGAGCTCGTGGTGG |
| YggS ΔK233/K234_for | TAAGAATTCGAGCTCCGTCGACAAGCTTGC |
| YggS ΔK233/K234_rev | CGGAGCTCGAATTCTTAGAGTAATCACGCGC |
| DH596 (BADyggS_fwd) | TATATACCATGGACGATATTGCGCATAACCTGGC |
| DH598 (BADyggS_rev) | TTAATTAAGCATGCTTATTTTTTAGAGTAATCACGCGC |
| JTBP94 (yggS-K36A fwd) | TGCAGTCAGTGCAACAAAACCTG |
| JTBP95 (yggS-K36A rev) | AGCAGCGTAATTTCTTCTG |
| JTBP98 (yggS-K38A fwd) | CAGTAAAACAGCACCTGCGAGCG |
| JTBP99 (yggS-K38A rev) | ACTGCAAGCAGCGTAATT |
| JTBP100 (yggS-K137A fwd) | TGAAAACAGTGCGTCCGGGATTC |
| JTBP101 (yggS-K137A rev) | TCACTAATGTTAATTTGAATCAG |
| JTBP102 (yggS-K36A/K38A fwd) | TGCAGTCAGTGCAACAGCACCTG |
| JTBP103 (yggS-K36A/K38A rev) | AGCAGCGTAATTTCTTCTG |
| JTBP106 (yggS-K233A fwd) | TGATTACTCTGCAAAATAAGCATGCAAGC |
| JTBP107 (yggS-K233A rev) | CGCGCACCAAAAATTGCA |
| JTBP108 (yggS-K234A fwd) | TTACTCTAAAGCATAAGCATGCAAGCTTG |
| JTBP109 (yggS-K234A rev) | TCACGCGCACCAAAAATT |
| JTBP110 (yggS-K233A/K234A fwd) | TGATTACTCTGCAGCATAAGCATGCAAGCTTGGC |
| JTBP111 (yggS-K233A/K234A fwd) | CGCGCACCAAAAATTGCA |

**Table S2.** Plasmids used for the expression of YggS variants

| **YggS forms** | **Plasmid** |
| --- | --- |
| WT YggS | pET28b(+) *yggS*-6xHis (Kan^R^) |
| WT YggS2 | pET28b(+) 6xHis-*yggS* (Kan^R^) |
| K36A | pET28b(+) *yggS*-6xHis K36A (Kan^R^) |
| K137A | pET28b(+) *yggS*-6xHis K137A (Kan^R^) |
| ΔK233/K234 | pET28b(+) 6xHis-*yggS* ΔK233/K234 (Kan^R^) |
| K36A/K137A | pET28(+) *yggS*-6xHis K36A/K137A (Kan^R^) |
| K36A/K233A/K234A | pET28(+) *yggS*-6xHis K36A/K233A/K234A (Kan^R^) |
| K36A/K38A | pET28(+) *yggS*-6xHis K36A/K38A (Kan^R^) |
| K36A/K137A/K233A/K234A | pET28(+) *yggS*-6xHis K36A/K137A/K233A/K234A (Kan^R^) |
| K36A/K38A/K137A | pET28(+) *yggS*-6xHis K36A/K38A/K137A (Kan^R^) |
| K36A/K38A/K233A/K234A | pET28(+) *yggS*-6xHis K36A/K38A/K233A/K234A (Kan^R^) |
| K36A/K38A/K137A/K233A/K234A | pET28(+) *yggS*-6xHis K36A/K38A/K137A/K233A/K234A (Kan^R^) |
| K38A/K137A/K233A/K234A | pET28b(+) *yggS*-6xHis K38A/K137A/K233A/K234A (Kan^R^) |

Kan^R^, kanamycin resistance

**Table S3.** YggS crystallization conditions

| **YggS** | **Protein Buffer**  **Solution** | **Crystallization conditions** |
| --- | --- | --- |
| Holo-YGGS | 0.05M K Phosphate,  0.15M NaCl, pH 7.5 | 0.1M Hepes/Na, pH7.5, 0.8M NaH_2_PO_4_/0.8M KH_2_PO_4_ |
| Apo-YGGS | 0.05M K Phosphate,  0.15M NaCl, pH 7.5 | 2M (NH_4_)_2_SO_4_, 5% Isopropanol |
| K36A | 0.05M K Phosphate,  0.15M NaCl, pH 7.5 | 0.1M Hepes/Na, pH7.5, 0.8M NaH_2_PO_4_/0.8M KH_2_PO_4_ |
| K137A | 0.05M K Phosphate,  0.15M NaCl, pH 7.5 | 1.1 M (NH_4_)_2_SO_4_, pH 8.5, 12% Glycerol |
| K38A/K137A/K233A/K234A | 0.05M K Phosphate,  0.15M NaCl, pH 7.5 | 0.1M Na Acetate, pH4.5, 20% Butanediol |
| K36A/K38A | 0.05M K Phosphate,  0.15M NaCl, pH 7.5 | 0.1M Hepes/Na, pH7.5, 0.8M NaH_2_PO_4_/0.8M KH_2_PO_4_ |
| K36A/K137A | 0.05M K Phosphate,  0.15M NaCl, pH 7.5 | 2M (NH_4_)_2_SO_4_, 0.1M CAPS/NaOH, pH 10.5, 0.2M Li_2_SO_4_ |
| K36A/K38A/K233A/K234A | 0.05M Tris-HCl, pH 7.2, 0.15M NaCl | 0.1M Na citrate/citric acid, pH4.0, 0.8M (NH_4_)_2_SO_4_ |
| YggS-PNP | 0.05M Na Hepes, pH7.5 | 35% Dioxane |

| Table S4. Plasmid constructs for *in vivo* 4dPN sensitivity assays of YggS-Lysine mutants | | | |
| --- | --- | --- | --- |
| **Construct ID** | **Plasmid** | **Template** | **Primers** |
| pBY291.3 | N/A – WT *yggS*  pBAD24::*yggS*_Ec_ (Amp^R^) | *E. coli* BW25113 | DH596/DH598 |
| pJTB1014 | pBAD24::*yggS* K36A (Amp^R^) | pBY291.3 | P94/P95 |
| pJTB1023 | pBAD24::*yggS* K38A (Amp^R^) | pBY291.3 | P98/P99 |
| pJTB1032 | pBAD24::*yggS* K137A (Amp^R^) | pBY291.3 | P100/P101 |
| pJTB1035 | pBAD24::*yggS* K36A/K137A (Amp^R^) | pJTB1014 | P100/P101 |
| pJTB1041 | pBAD24::*yggS* K233A (Amp^R^) | pBY291.3 | P106/P107 |
| pJTB1046 | pBAD24::*yggS* K36A/K233A (Amp^R^) | pJTB1014 | P106/P107 |
| pJTB1047 | pBAD24::*yggS* K234A (Amp^R^) | pBY291.3 | P108/P109 |
| pJTB1050 | pBAD24::*yggS* K36A/K234A (Amp^R^) | pJTB1014 | P108/P109 |
| pJTB1053 | pBAD24::*yggS* K36A/K38A (Amp^R^) | pJTB1023 | P102/P103 |
| pJTB1056 | pBAD24::*yggS* K38A/K233A (Amp^R^) | pJTB1023 | P106/P107 |
| pJTB1059 | pBAD24::*yggS* K38A/K234A (Amp^R^) | pJTB1023 | P108/P109 |
| pJTB1062 | pBAD24::*yggS* K233A/K234A (Amp^R^) | pJTB1041 | P110/P111 |
| pJTB1065 | pBAD24::*yggS* K38A/K233A/K234A (Amp^R^) | pJTB1059 | P110/P111 |
| pJTB1074 | pBAD24::*yggS* K38A/K137A (Amp^R^) | pJTB1023 | P100/P101 |
| pJTB1079 | pBAD24::*yggS* K38A/K137A/K233A/K234A (Amp^R^) | pJTB1065 | P100/P101 |
| pJTB1080 | pBAD24::*yggS* K36A/K38A/K137A (Amp^R^) | pJTB1053 | P100/P101 |
| pJTB1082 | pBAD24::*yggS* K36A/K38A/K233A/K234A (Amp^R^) | pJTB1065 | P102/P103 |
| pJTB1084 | pBAD24::*yggS* K36A/K233A/K234A (Amp^R^) | pJTB1062 | P94/P95 |
| pJTB1086 | pBAD24::*yggS* K137A/K233A/K234A (Amp^R^) | pJTB1062 | P100/P101 |
| pJTB1088 | pBAD24::*yggS* K36A/K38A/K137A/K233A/K234A (Amp^R^) | pJTB1080 | P110/P111 |
| pJTB1091 | pBAD24::*yggS* K36A/K137A/K233A/K234A (Amp^R^) | pJTB1084 | P100/P101 |

Amp^R^, Ampicillin resistance


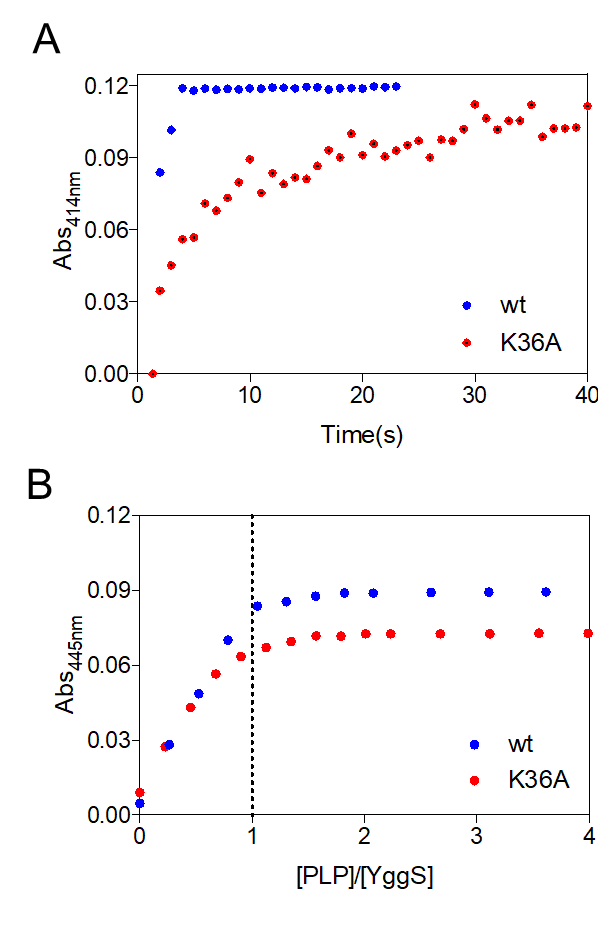


**Figure S1.** A) Time course of Schiff base formation observed upon mixing 20 µM WT YggS (blue symbols) and K36A variant (red symbols) with 50 µM free PLP, monitored by measuring the absorbance at 415 nm, which corresponds to the maximum absorbance wavelength of the protonated Schiff base. B) Titration of PLP binding through Schiff base formation was carried out by the addition of increasing concentrations of PLP to WT YggS (blue symbols) and K36A variant (red symbols) (20 µM each). Formation of protonated Schiff base was monitored at 445 nm, where absorbance of free PLP was negligible.


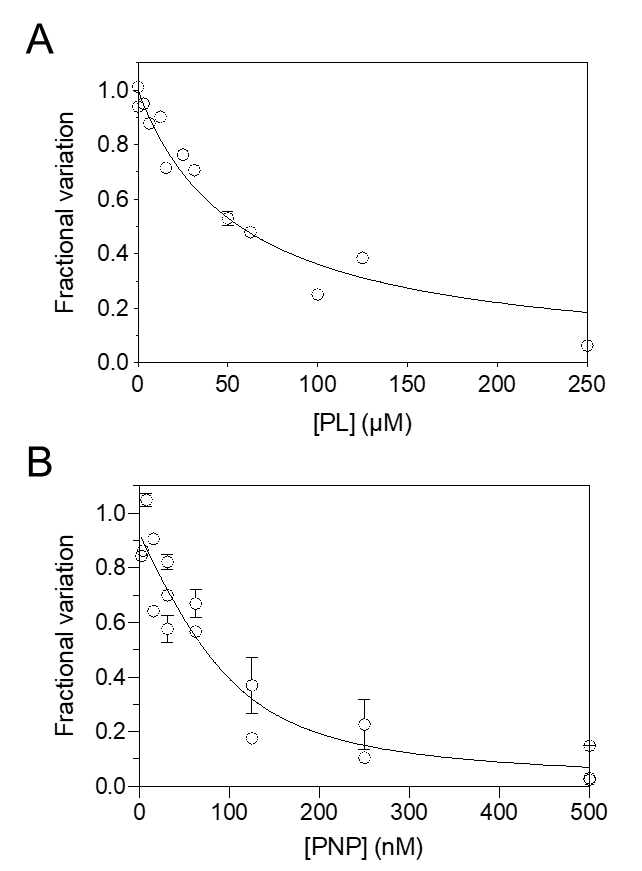


**Figure S2.** PL and PNP binding equilibria. Fluorescence emission (expressed as fractional variation) between 315 and 325 nm as a function of total PL (A) and PNP (B) concentration.


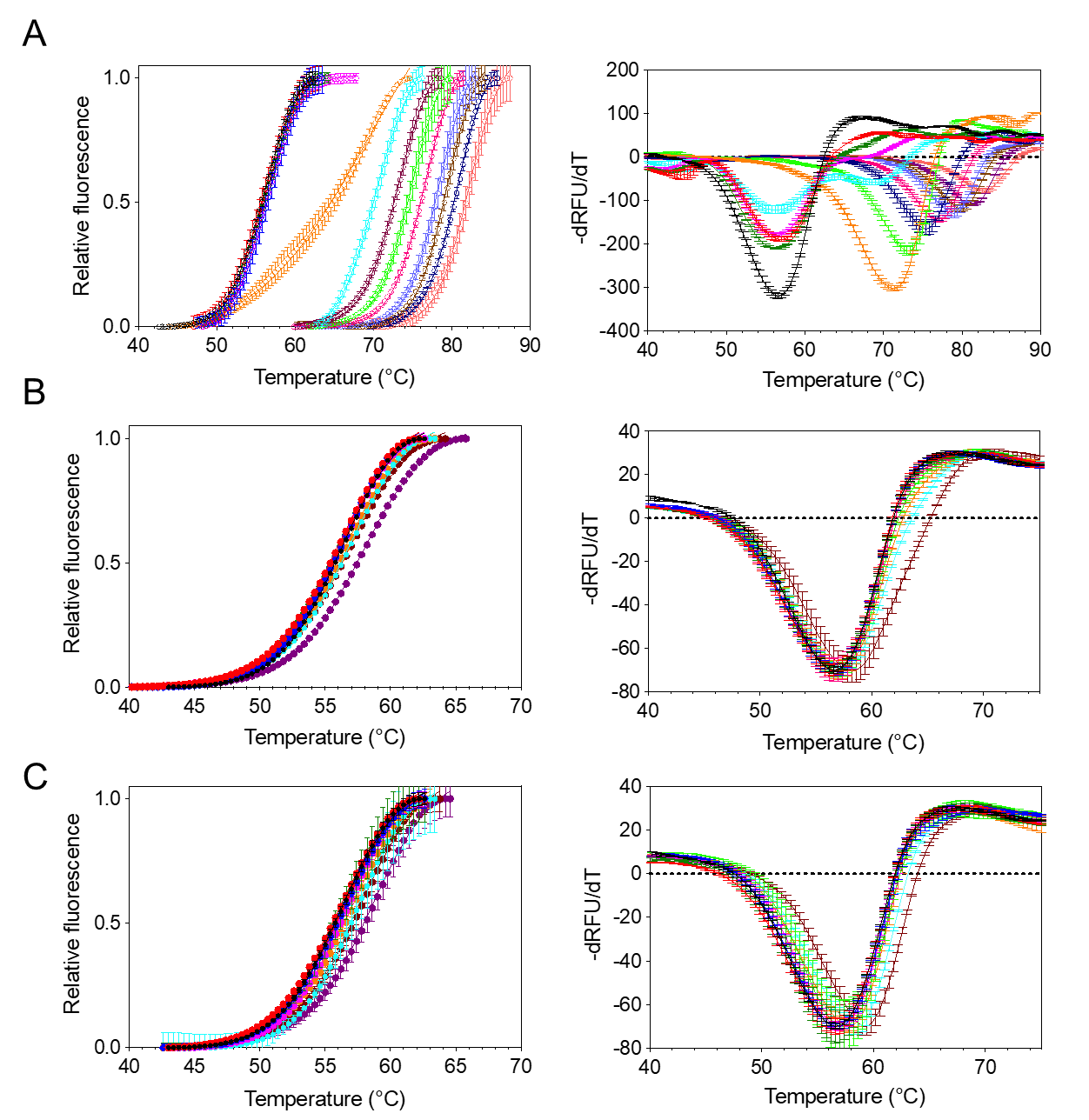


**Figure S3.** DSF measurements of apo-YggS in the presence of different concentrations of PLP (A), PL (B) and PNP (C)*.* Left panels*:* fluorescence change expressed as fractional variation as a function of temperature. The experiment was carried out using 2 µM protein and different vitamer concentrations (0, 0.29, 0.59, 1.17, 2.34, 4.69, 9.38, 18.75, 37.5, 75, 150 and 300 µM). Thermal denaturation data were fitted to the Boltzmann equation to obtain melting temperatures. Each curve is the average of three independent experiments. Right panels: first derivative (-dF/dT) of denaturation profiles showed in the left panels.


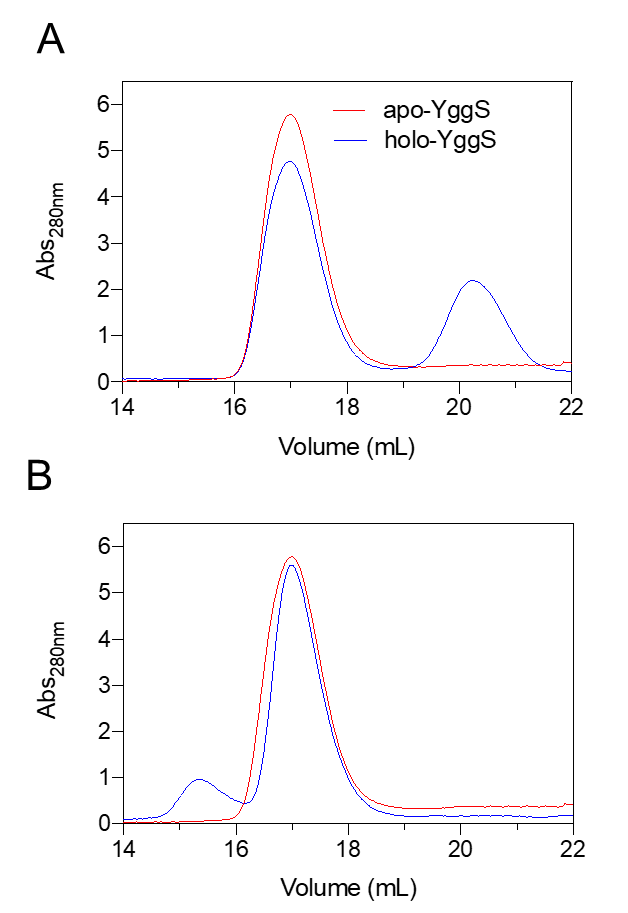


**Figure S4.** A. and B. Size exclusion chromatography analysis of WT YggS. Chromatographic runs were performed on a Superdex 200 10/300 GL column (GE Healthcare) in 50 mM NaHEPES, pH 7.6. A) Elution profiles obtained with apo- (red line) and holo-YggS (blue line, with excess free PLP eluting at 20.3 mL) that were purified in the presence of 2.5 mM 2-mercaptoethanol. B) Elution profiles of holo-YggS purified in the presence (red line) or absence (blue line) of 2.5 mM 2-mercaptoethanol.


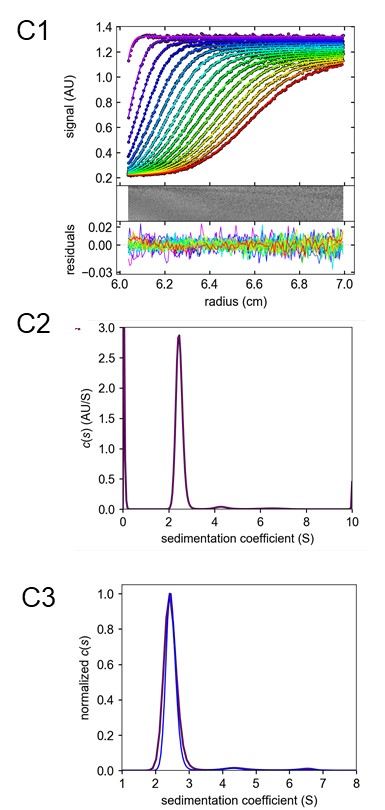


**Figure S4.** C. AUC analyses of WT YggS. C1) Raw sedimentation scan at 280 nm of WT YggS. C2) Sedimentation coefficient distribution of Ni-NTA purified WT YggS. C3) Normalized plot of the interference and absorbance molecular distributions, revealing mainly 29.1 kDa monomer.


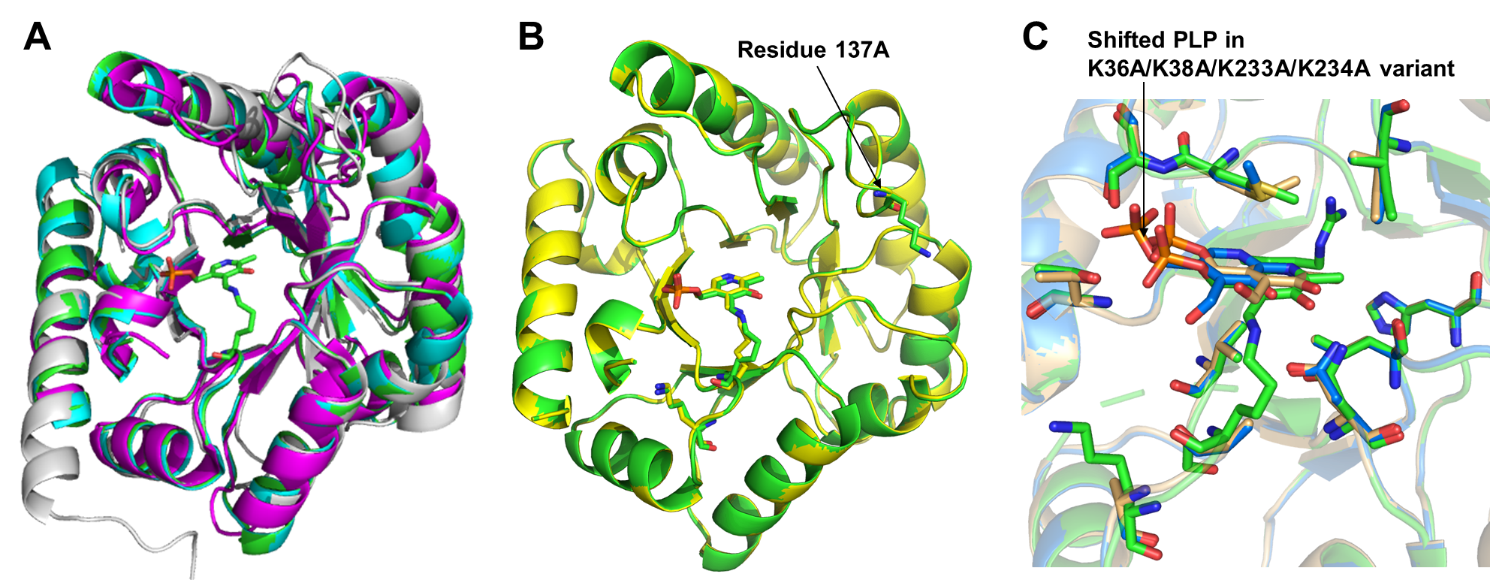


**Figure S5**. Superposition of holo-YggS (green) with 1W8G (cyan), 5NM8 (magenta) and 1CT5 (grey).

**Figure S6.**  Multiple alignment of YggS from eukaryotic and prokaryotic sources obtained using the Clustal Omega program (<https://www.ebi.ac.uk/Tools/msa/clustalo/>) provided by the EMBL's European Bioinformatics Institute. The *E. coli* YggS lysine residues K36, K38, K65, K89, K137, K233 and K234 and the corresponding invariant lysine residues of YggS from other sources are in red. Positively charged residues occupying either the same or adjacent positions are highlighted in red. Sources of the aligned YggS amino acid sequences and related accession numbers are: *Escherichia coli* (E.col), (WP_001603486.1); *Fusobacterium nucleatum* (F.nuc), (WP_249527286.1); *Bifidobacterium adolescentis* (B.ado), (WP_003810092.1); *Agrobacterium tumefaciens* (A.tum), (WP_035258020.1), *Synechococcus elongatus* (S.elo), (WP_011244343.1); *Homo sapiens* (H.sap), (NP_009129.1); *Agrobacterium tumefaciens* (A.tum), (WP_035258020.1); *Saccharomyces cerevisiae* S288C (S.cer), (NP_009517.1); *Arabidopsis thaliana* x *Arabidopsis arenosa* (A.tha), (KAG7591360. 1).


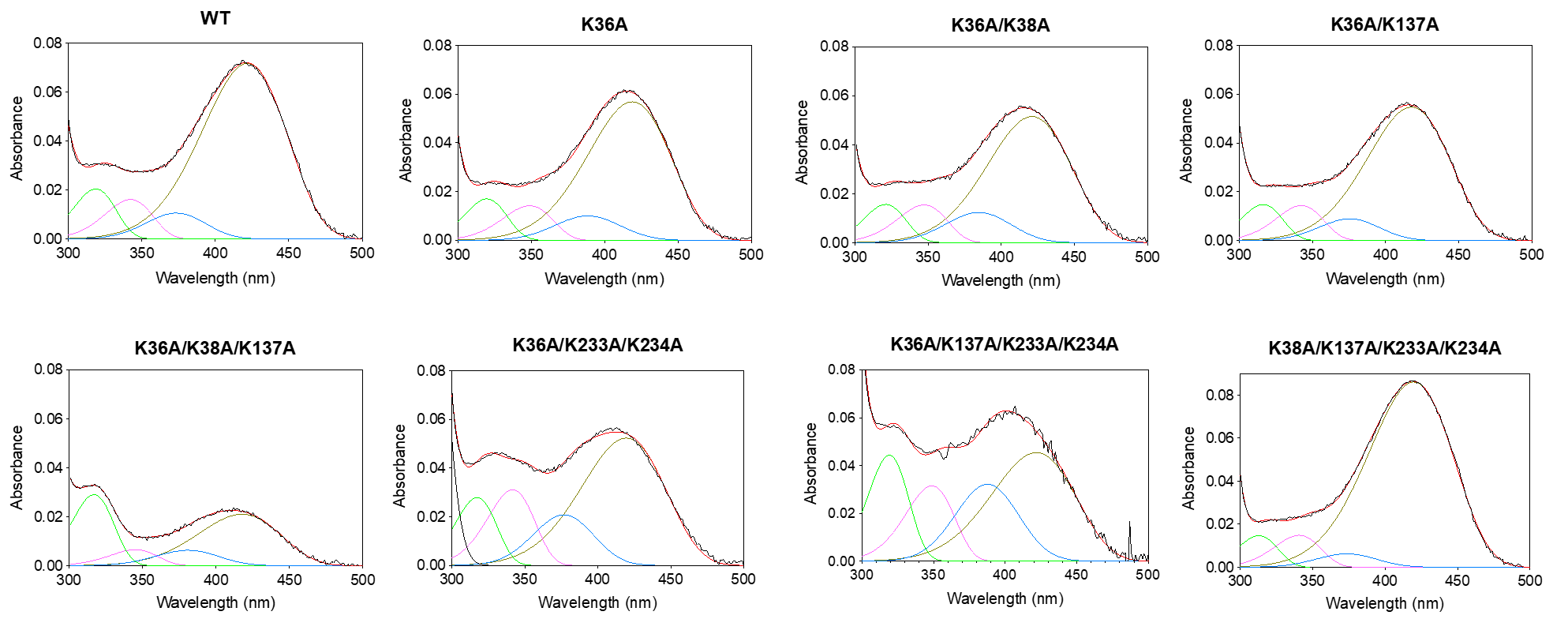


**Figure S7.** Deconvolution of absorption spectra of the indicated YggS forms, carried out as described in Materials and methods. The noisy black lines are the experimentally measured absorption spectra, while the red lines correspond to the sum of the component bands obtained from the deconvolution procedures (colored lines).

**Figure S8.** Absorption spectra of protein samples (blue and red continuous lines correspond to WT and K36A YggS samples, respectively) and small molecular weight samples (dashed lines, same color code) after reduction with NaBH_4_, denaturation with NaOH and filtering, as described in the main text of the manuscript.


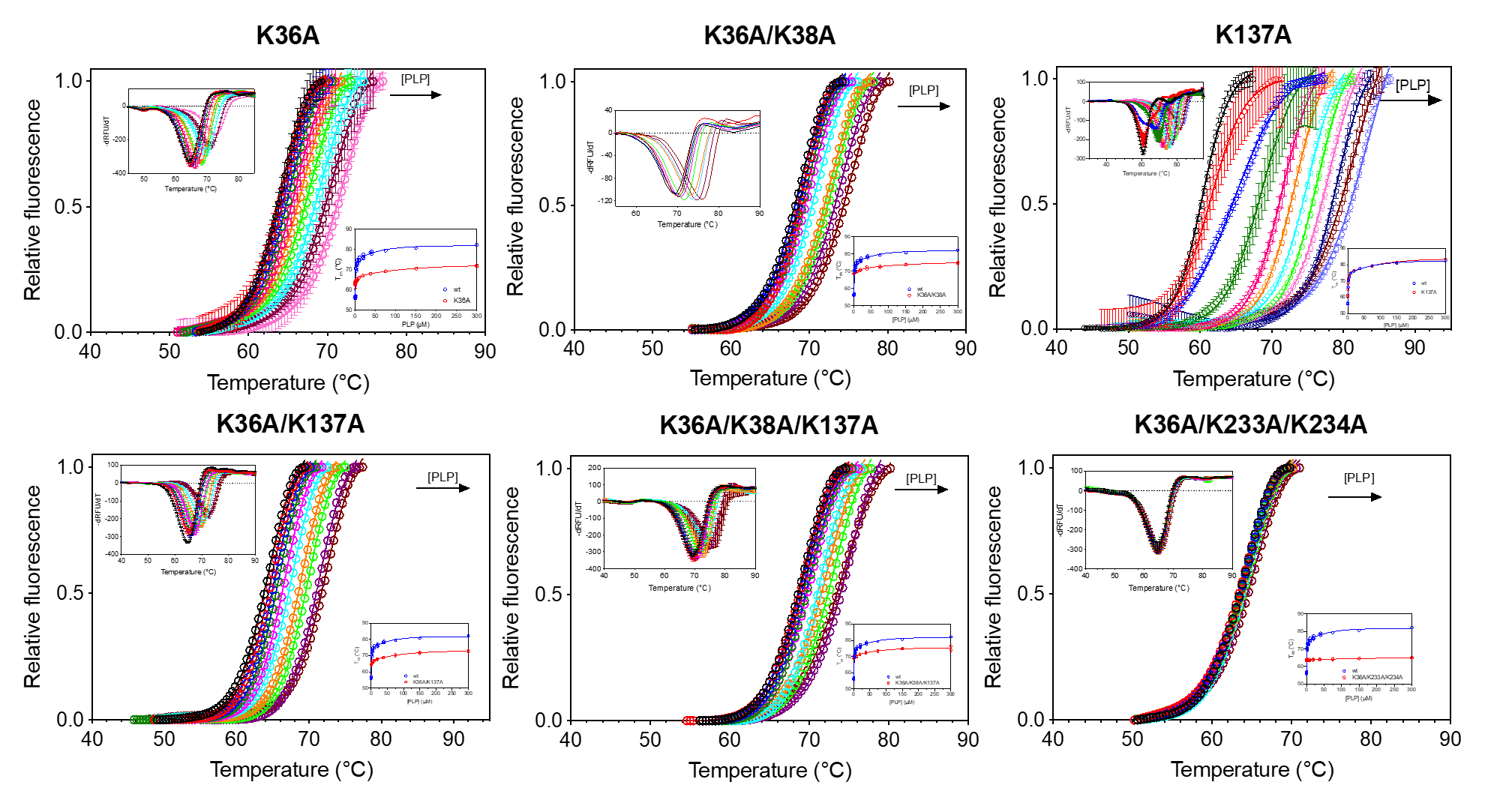


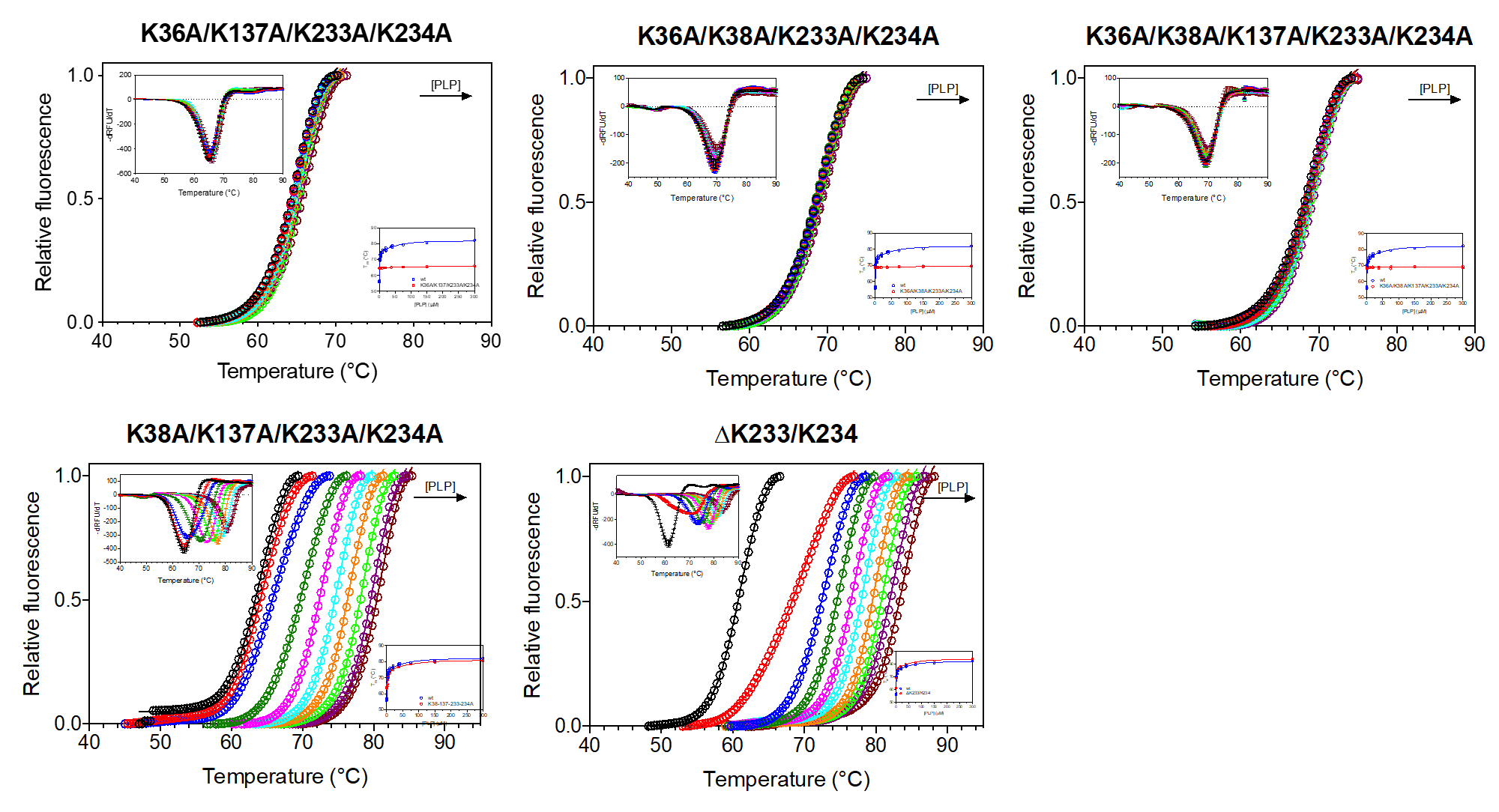


**Figure S9.** DSF measurements of apo-YggS variants in the presence of different concentrations of PLP*.* The fluorescence change is expressed as fractional variation as a function of temperature. The experiment was carried out using 2 µM enzyme and different PLP concentrations (0, 0.29, 0.59, 1.17, 2.34, 4.69, 9.38, 18.75, 37.5, 75, 150 and 300 µM). Thermal denaturation data were fitted to the Boltzmann equation to obtain melting temperatures. Each curve is the average of three independent experiments. Upper insets: first derivative (-dF/dT) of denaturation profiles showed in the main panels. Lower insets: Variation of the melting temperatures, obtained by DSF analysis, as a function of PLP concentration.


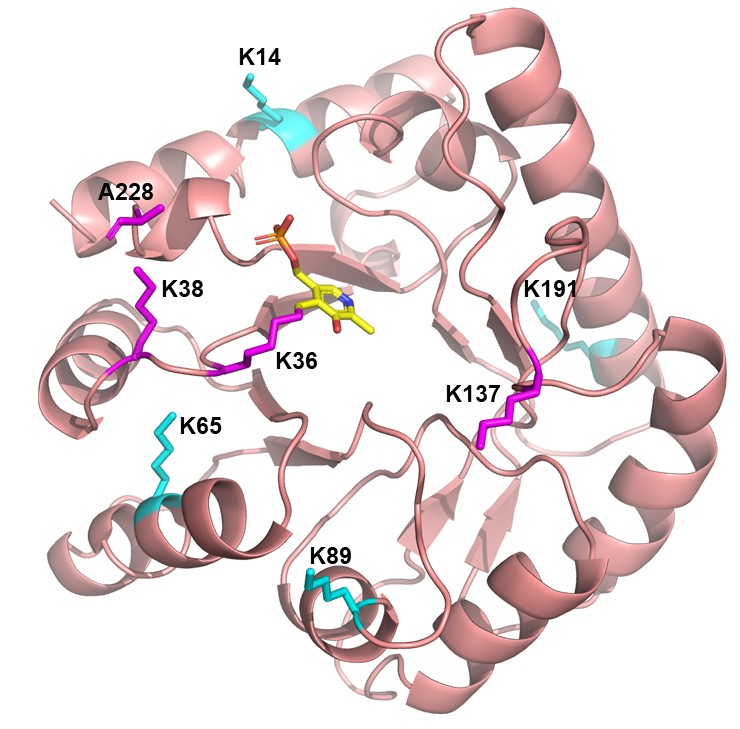


**Figure S10.** Cartoon representation of WT holo-YggS crystal structure solved and described in this work (PDB ID: 7U9C). The mutated lysine residues are represented as magenta sticks (K36, K38 and K137). Since C-terminal residues 229-234 are not visible in the electron density map, lysines K233 and K234 could not be represented, however, the A228 residue is shown as magenta sticks to approximately locate the C-terminal end of the protein. Other lysine residues are represented as cyan sticks. PLP is shown as yellow sticks.


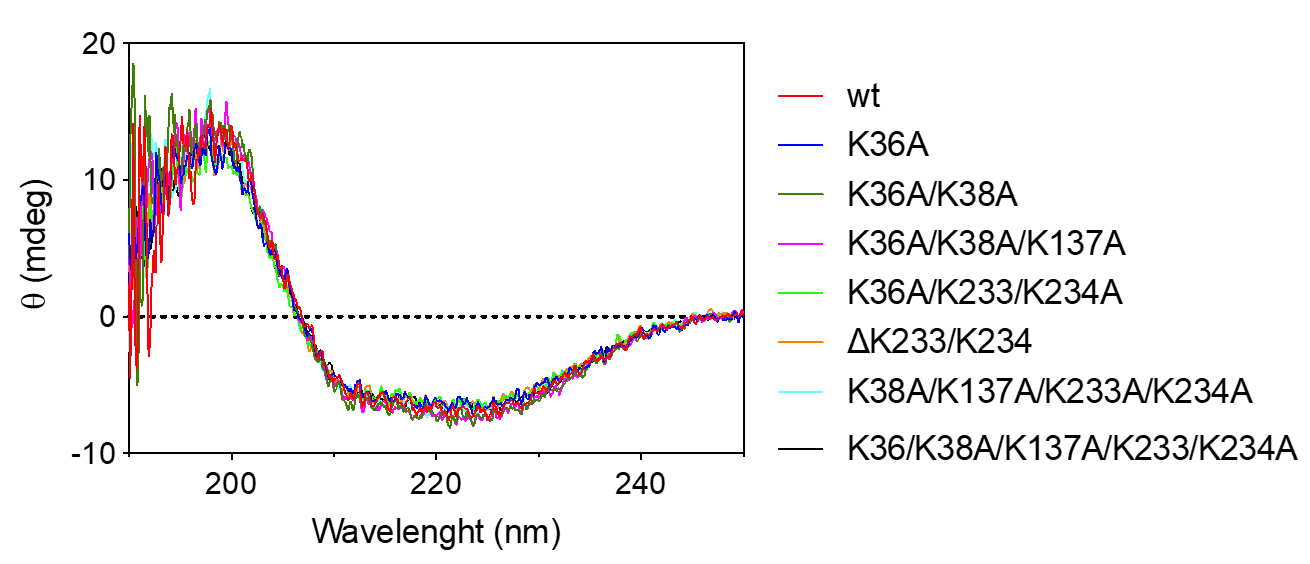


**Figure S11.** Far-UV CD spectra of WT and variant YggS forms. CD spectra were measured in 50 mM NaHEPES buffer, pH 7.6.

A

**
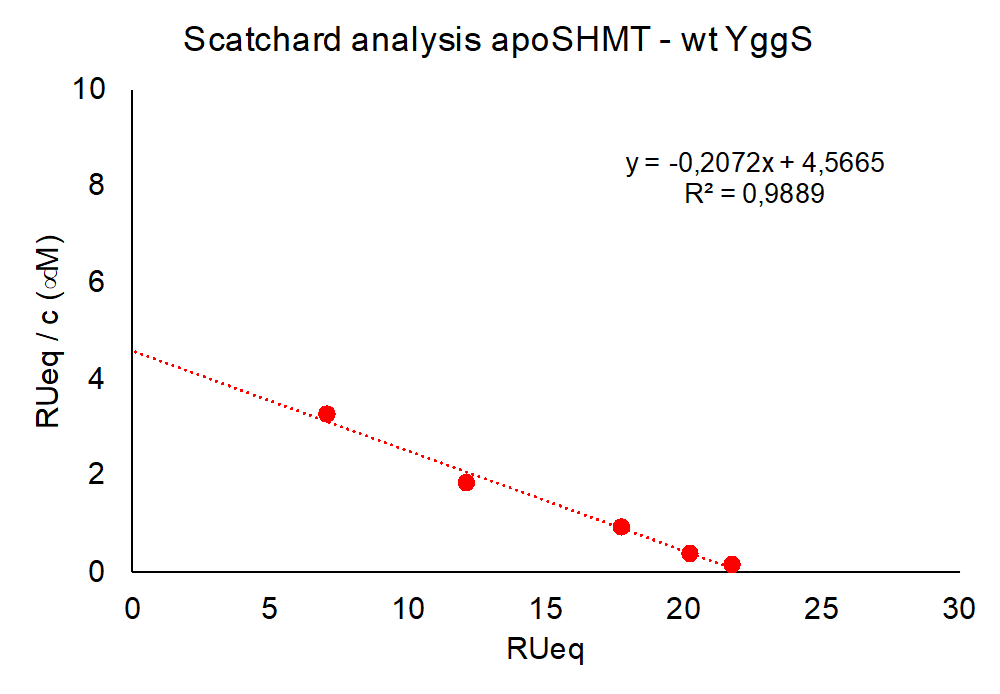
**

B

**Figure S12.** SPR analyses of YggS-SHMT interactions. (A) Sensorgrams measured injecting wild-type YggS at the following concentrations: 0.24, 0.72, 2.2, 6.7, 20, 60, and 180 μM. (B) Scatchard analysis of sensorgrams: wild-type YggS (red circles) interacts with apoSHMT with a K_D_ of 4.8 ± 0.8 μM.


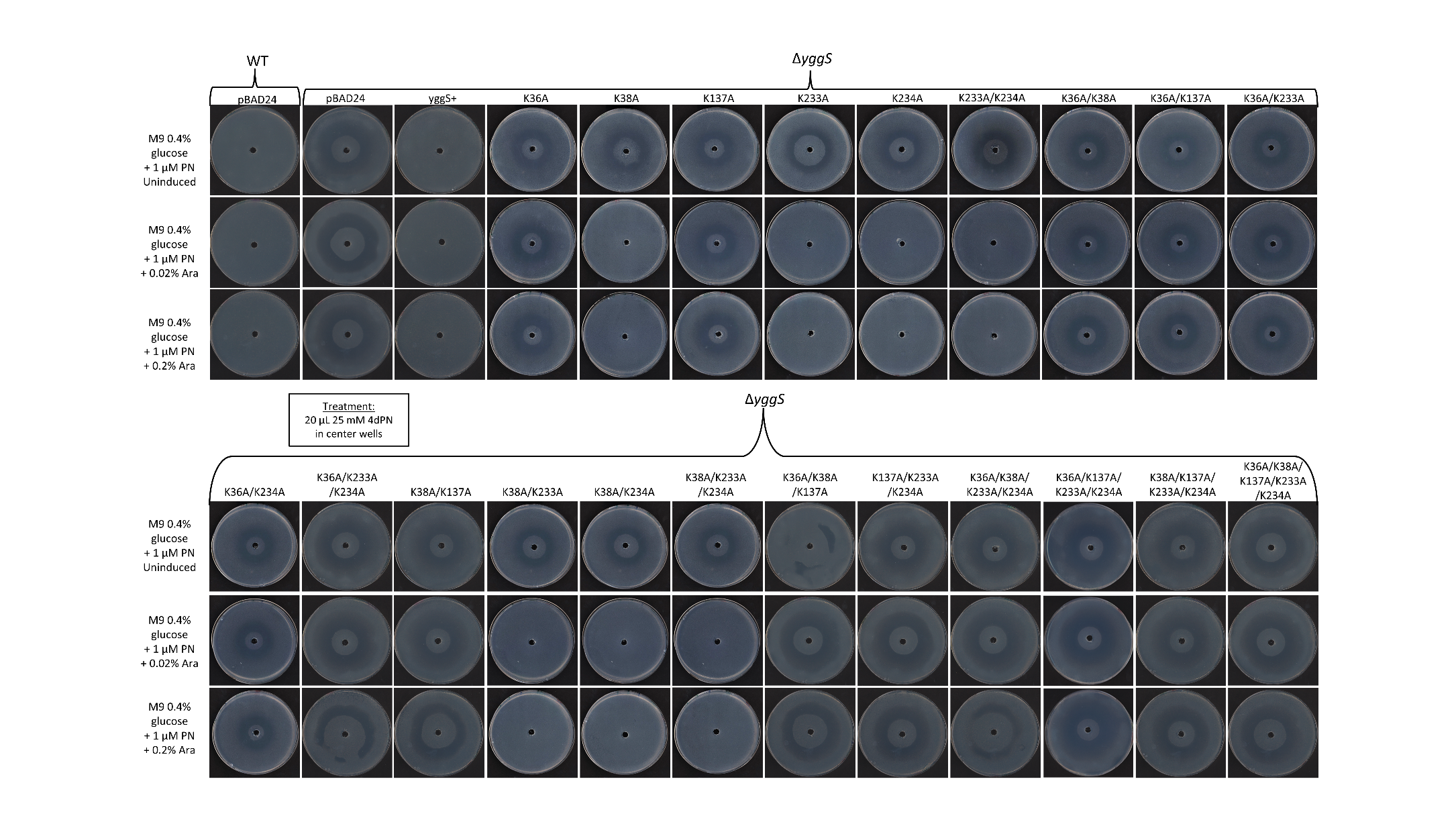


**Figure S13**. *In vivo* complementation of the 4-deoxypyridoxine (4dPN) sensitivity phenotype by expression of all YggS lysine variants *in trans*. Experiments were carried out as described in the methods section and in Figure 8.


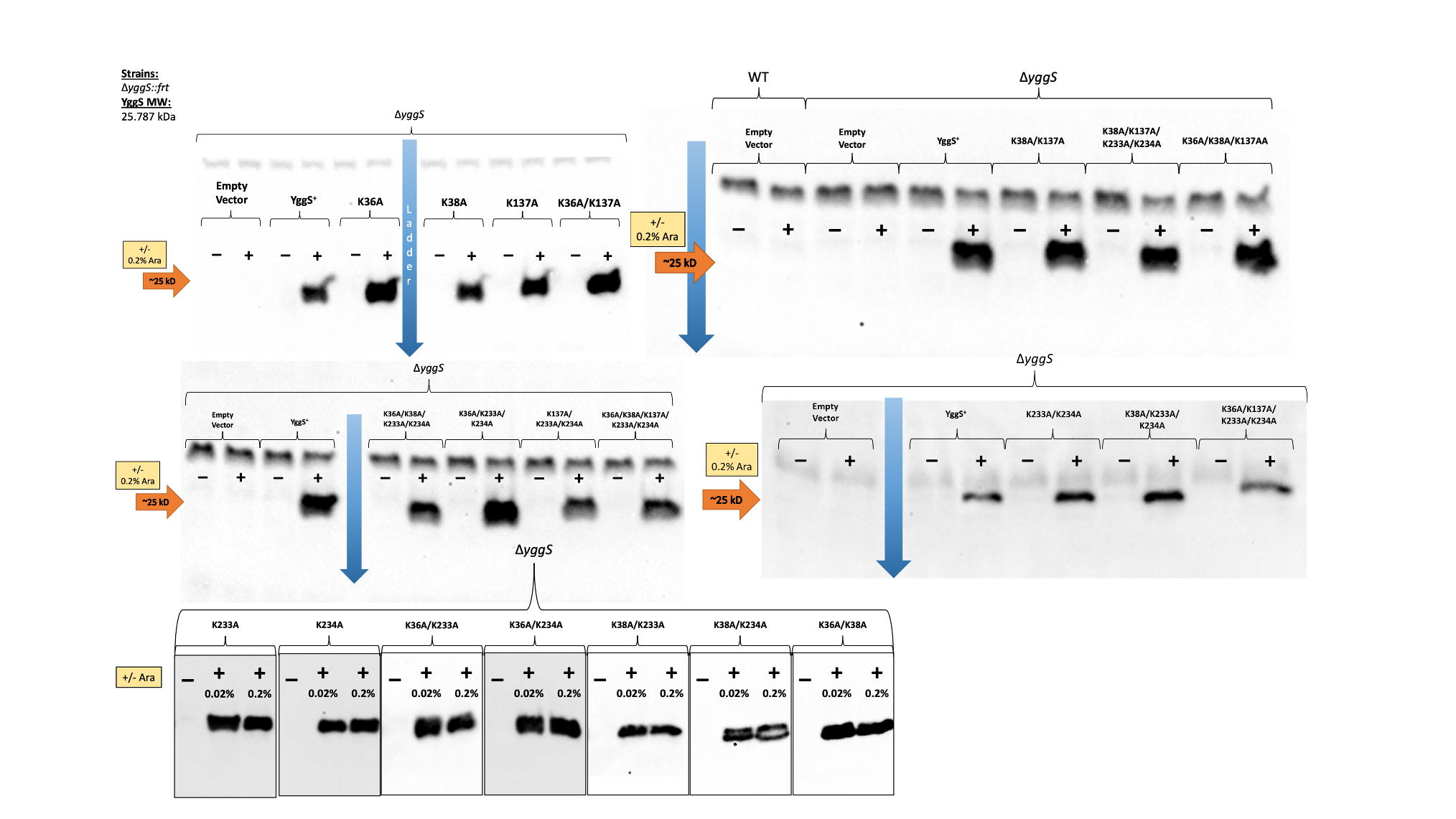


**Figure S14**. Western blots for verification of functional protein expression of WT- YggS and YggS-Lysine mutant constructs *in trans.* Midlog cells of experimental cultures grown with or without arabinose induction were collected and normalized for western blot analysis as described in the methods section. Samples were taken from experimental cultures used in 4dPN sensitivity assays to ensure expression and validity of each experiment.
